# The study of the determinants controlling Arpp19 phosphatase-inhibitory activity reveals an Arpp19/PP2A-B55 feedback loop

**DOI:** 10.1038/s41467-021-23657-0

**Published:** 2021-06-11

**Authors:** Jean Claude Labbé, Suzanne Vigneron, Francisca Méchali, Perle Robert, Sylvain Roque, Cindy Genoud, Perrine Goguet-Rubio, Phillipe Barthe, Gilles Labesse, Martin Cohen-Gonsaud, Anna Castro, Thierry Lorca

**Affiliations:** 1 Université de Montpellier, Centre de Recherche en Biologie Cellulaire de Montpellier (CRBM) CNRS, UMR 5237, Montpellier, France; 2 Équipe Labellisée “Ligue Nationale Contre le Cancer”, Montpellier, France; 3 Centre de Biologie Structurale (CBS), CNRS, INSERM, Université de Montpellier, Montpellier, France

**Keywords:** Cell division, Meiosis, Mitosis

## Abstract

Arpp19 is a potent PP2A-B55 inhibitor that regulates this phosphatase to ensure the stable phosphorylation of mitotic/meiotic substrates. At G2-M, Arpp19 is phosphorylated by the Greatwall kinase on S67. This phosphorylated Arpp19 form displays a high affinity to PP2A-B55 and a slow dephosphorylation rate, acting as a competitor of PP2A-B55 substrates. The molecular determinants conferring slow dephosphorylation kinetics to S67 are unknown. PKA also phosphorylates Arpp19. This phosphorylation performed on S109 is essential to maintain prophase I-arrest in Xenopus oocytes although the underlying signalling mechanism is elusive. Here, we characterize the molecular determinants conferring high affinity and slow dephosphorylation to S67 and controlling PP2A-B55 inhibitory activity of Arpp19. Moreover, we show that phospho-S109 restricts S67 phosphorylation by increasing its catalysis by PP2A-B55. Finally, we discover a double feed-back loop between these two phospho-sites essential to coordinate the temporal pattern of Arpp19-dependent PP2A-B55 inhibition and Cyclin B/Cdk1 activation during cell division.

## Abstract (web summary)

Progression of the cell division cycle requires feedback loops including those of phosphorylation and dephosphorylation; however the precise regulation of phosphorylation kinetics of Arpp19, an inhibitor of protein phosphatase 2A, is unclear. Here, the authors report that feedback between phosphorylation states of Ser67 and Ser109 of Arpp19 coordinates Arpp19-dependent inhibition of PP2A-B55 and Cyclin B activation during cell cycle progression.

## Introduction

Entry and exit of mitosis and meiosis is induced by oscillations of protein phosphorylation/dephosphorylation. These oscillations are the result of the activation and inactivation of the master kinase Cyclin B/Cdk1 and of its counterbalancing phosphatase PP2A-B55^1–7^. Although substrate phosphorylation is triggered at mitotic entry by the activation of Cyclin B/Cdk1, it is only fully achieved if PP2A-B55 activity is negatively regulated ^8–11^. This regulation is in charge of Arpp19 and ENSA, two members of the endosulfine family of proteins recently identified as two potent inhibitors of PP2A-B55^3,4^. PP2A-B55 inhibition does not only control mitosis but also other phases of the cell cycle. In this line, ENSA negatively regulates this phosphatase during DNA replication and promotes the dephosphorylation and degradation of the replication factor Treslin^12^. Conversely, Arpp19 is an essential gene controlling PP2A-B55 during mitotic division. Indeed, the ablation of this protein in mouse embryonic fibroblasts promotes the premature dephosphorylation of mitotic substrates resulting in the disruption of the correct temporal order of cellular events during mitotic progression^6^. Both Arpp19 and ENSA are the unique substrates of the Greatwall (Gwl) kinase. At G2-M onset, Gwl is activated and phosphorylates Arpp19 at a single site, S67. Arpp19 phosphorylation then triggers its binding and the subsequent inhibition of PP2A-B55, hence allowing the stable phosphorylation of Cyclin B/Cdk1 substrates and mitotic entry^5,13^. At mitotic exit, Gwl is inactivated, resulting in Arpp19 dephosphorylation, PP2A-B55 reactivation and the gradual dephosphorylation of mitotic substrates^14,15^. The mechanisms by which Arpp19/ENSA inhibit PP2A-B55 are still elusive; however, a previous report demonstrated that ENSA acts as a competitive substrate of PP2A-B55^16^. Accordingly, data established that phospho-S67 ENSA displays a high affinity for this phosphatase but a slow dephosphorylation rate and consequently acts as a major competitor of PP2A-B55 substrates. The molecular determinants of Arpp19 and ENSA conferring these specific properties to phospho-S67 are completely unknown.

Besides Gwl-dependent phosphorylation, Arpp19 is also phosphorylated by protein kinase A (PKA). PKA-dependent phosphorylation of the alternative splice variant of Arpp19, the protein Arpp16, was first described. This phosphorylation promotes Arpp16 binding and inhibition of PP2A-B55, a regulation that is essential for striatal cell signalling^17^. Besides Arpp16, Arpp19 is also phosphorylated by PKA in *Xenopus* oocytes. PKA-dependent phosphorylation of Arpp19 on residue S109 is essential to arrest *Xenopus* oocytes in prophase I of meiosis^18^. Accordingly, the injection of a S109D phospho-mimetic Arpp19 form to these oocytes blocks meiotic resumption induced by progesterone (PG). How phospho-S109 Arpp19 negatively regulates meiotic resumption is completely unknown.

In this work, we identify the major molecular determinants controlling the PP2A-B55 inhibitory activity of Arpp19. Moreover, we elucidate the mechanisms by which phospho-S109 controls S67 Arpp19 phosphorylation and, thus, PP2A-B55 inhibitory activity. Finally, we have discovered a double feedback loop between these two phospho-sites, which would be required to coordinate the proper temporal pattern of Arpp19-dependent PP2A-B55 inhibition and Cyclin B/Cdk1 activation, hence ensuring a correct progression through meiosis and mitosis.

## Results

### DSG residues control Arpp19 dephosphorylation and PP2A-B55 binding

Arpp19 is an intrinsically disordered protein that potently inhibits PP2A-B55. Despite its major role in the control of cell cycle progression^3–6,19^, little is known about the mechanisms by which this protein modulates phosphatase activity. The binding and the inhibition of PP2A-B55 by Arpp19 require its phosphorylation at S67 by Gwl. This site is located in a motif highly conserved from yeast to humans (QKYFDSGDY), which we will refer to from now as the DSG motif^3,16,20,21^. Once phosphorylated, Arpp19 acts as a highly competitive substrate that tightly binds PP2A-B55 but is slowly dephosphorylated by this phosphatase^16^. Why S67 dephosphorylation of Arpp19 is so slow is unknown. We sought to determine the properties contributing to the inhibitory activity of Arpp19. To this end, we used the well established cell-free extract system of *Xenopus* oocytes. In this model, we measured the three major properties of this inhibitor as follows: (1) Gwl site dephosphorylation kinetics, (2) binding to PP2A-B55 and (3) physiologic capacity to promote mitotic entry.

Previous data propose that the dephosphorylation of ENSA, another member of the endosulfine family, is modulated by the presence of basic KR residues flanking the DSG motif. These residues would be a PP2A-B55 recognition signal that would promote binding to PP2A-B55 and would ensure timely ENSA dephosphorylation^22^.

**Fig. 1:**
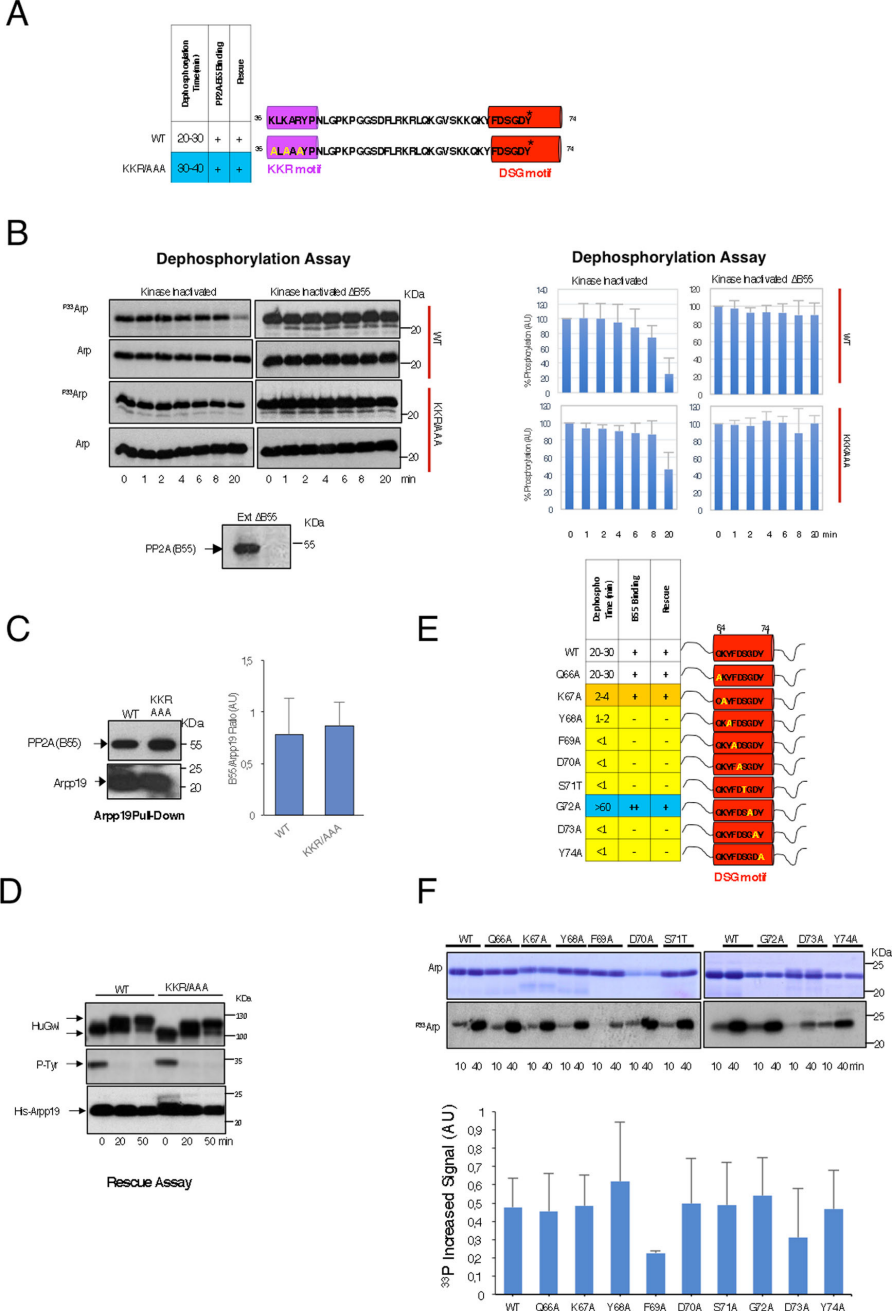
Basic residues close to the DSG motif positively modulate S67/S71 dephosphorylation of Arpp19. **A** DSG (red) and the KKR (violet) motifs, and the triple K36A/K38A/R40A (KKR/AAA) *Xenopus* Arpp19 alanine mutant. Table depicting dephosphorylation time, B55-Arpp19 interaction and the capacity to promote mitotic entry in Arpp19-depleted extracts. **B** 50 ng of wild type or KKR/AAA Arpp19 mutant phosphorylated ‘in vitro’ by GwlK72M was supplemented to kinase-inactivated extracts depleted or not of the B55 protein. Arpp19 levels and of S67/S71 phosphorylation were analysed by western blotting and autoradiography, respectively. The ^33^P-Arpp19/western blotting Arpp19 signal ratios were calculated using ImageJ for each time point. The percentage of the ratio remaining at each time point with respect to the ratio at 0 min was calculated and represented in bar graphs as the mean percentage ± SD; *n* = 3 biological independent samples. **C** A His-Arpp19 pulldown equivalent to 20 ng of wild type or KKR/AAA mutant was submitted to western blotting, and the amount of B55 and the levels of Arpp19 bound to the beads shown. B55/Arpp19 signal ratios were calculated using ImageJ and represented in a bar graph as the mean ratio ± SD; *n* = 5 biological independent samples. **D** Arpp19-depleted extracts were supplemented with human GwlK72M and a wild type or a KKR/AAA Arpp19 mutant and phosphorylation of Human Gwl, of Tyr15, of Cdk1 and Arpp19 ectopic levels (His-Arpp19) were assessed. **E** A schematic of DSG regions indicating residues mutated into alanine or threonine. Table representing results on the S67/S71 dephosphorylation time and the capacities to bind B55 or to restore mitotic entry in Arpp19-depleted egg extracts of each Arpp19 form. **F** Wild-type Arpp19 and the indicated mutants of the DSG motif were ‘in vitro’ phosphorylated by hGwlK72M and 1 µl sample removed at 10 and 40 min, to measure S67/S71 phosphorylation by autoradiography (^P33^Arp). The amount of Arpp19 was assessed by Coomassie blue staining (Arp)^33^. P-Arpp19 levels were normalized by Arpp19 amount using Coomassie blue signal and the increase in phosphorylation between time 10 and 40 min calculated. Data from three different experiments was then used to obtain the mean ± SD and represented in a bar graph; *n* = 3 biological independent samples.

To determine whether dephosphorylation and binding of Arpp19 to PP2A-B55 are modulated by basic KR residues, we mutated into alanine three of the basic aminoacids flanking the DSG motif of Arpp19 isoform 1 cloned from Stage VI oocytes. This isoform displays four additional aminoacids at positions 62–65 and, consequently, residue numbering will differ accordingly in the present study. We constructed a triple alanine mutant of the K36/K38/R40 region (named hereafter as the KKR motif) (Fig. 1A), which was further phosphorylated ‘in vitro’ by recombinant Gwl and γ^P33^ATP. Phosphorylated wild type and triple KKR mutant Arpp19 were then supplemented to interphase *Xenopus* egg extracts that were devoid of ATP to maintain kinases inactive (hereafter called kinase-inactivated extracts). The dephosphorylation pattern of the Gwl site S67 (S71 in Arpp19 isoform 1) was then followed by autoradiography to directly measure phosphatase activity and the levels of Arpp19 by western blotting (Fig 1B and Supplementary Fig. 1A). S67/S71 dephosphorylation of wild-type Arpp19 started at 8 min upon its addition to kinase-inactivated extracts, gradually decreased and fully disappeared at 30 min (Fig. 1B and Supplementary Fig. 1A). This dephosphorylation is catalysed by PP2A-B55, as it was fully blocked upon B55 depletion (Fig. 1B). Dephosphorylation of S67/S71 in the K36A/K38A/R40A (KKR/AAA) triple mutant was moderately delayed compared to wild-type Arpp19 and a complete dephosphorylation was only observed at 40 min (Fig. 1B and Supplementary Fig. 1A). This data indicates that these basic residues would positively modulate the dephosphorylation of S67/S71 on Arpp19.

We next checked whether, as previously proposed, the loss of KKR residues could delay S67/S71 Arpp19 dephosphorylation by decreasing its interaction to PP2A-B55^22^. However, PP2A-B55 association was not modified compared to wild-type Arpp19 (Fig. 1C). As so, this mutant kept full PP2A-B55 inhibitory capacity and promoted mitotic entry when added to Arpp19-depleted egg extracts as indicated by Gwl phosphorylation and tyrosine 15 Cdk1 dephosphorylation (Fig. 1D).

We subsequently investigated the impact of mutating Arpp19 in the DSG highly conserved motif. Each amino acid of this motif was mutated into alanine, except for the Gwl site S67/S71 whose alanine mutation results in a complete loss of PP2A-B55 binding^3^. Instead, we substituted this site by threonine, an amino acid to which PP2A-B55 displays an inherent preference^23^ (Fig. 1E). We could not find differences in the capacity of a purified Gwl form to phosphorylate S67/S71 ‘in vitro’ in most of the mutants, except for F69A whose phosphorylation was fairly decreased (Fig. 1F). This data disagrees with a previous report suggesting a dramatic drop in the phosphorylation of this site in ENSA by Gwl when aromatic and acidic residues, as well as S67/S71 itself, were mutated in human cells^22^. We do not know the reason for these differences. However, it is possible that the decline of S67/S71 phosphorylation observed in these ENSA mutants could result from increased PP2A-B55-dependent dephosphorylation instead of decreased Gwl-dependent phosphorylation.

**Fig. 2:**
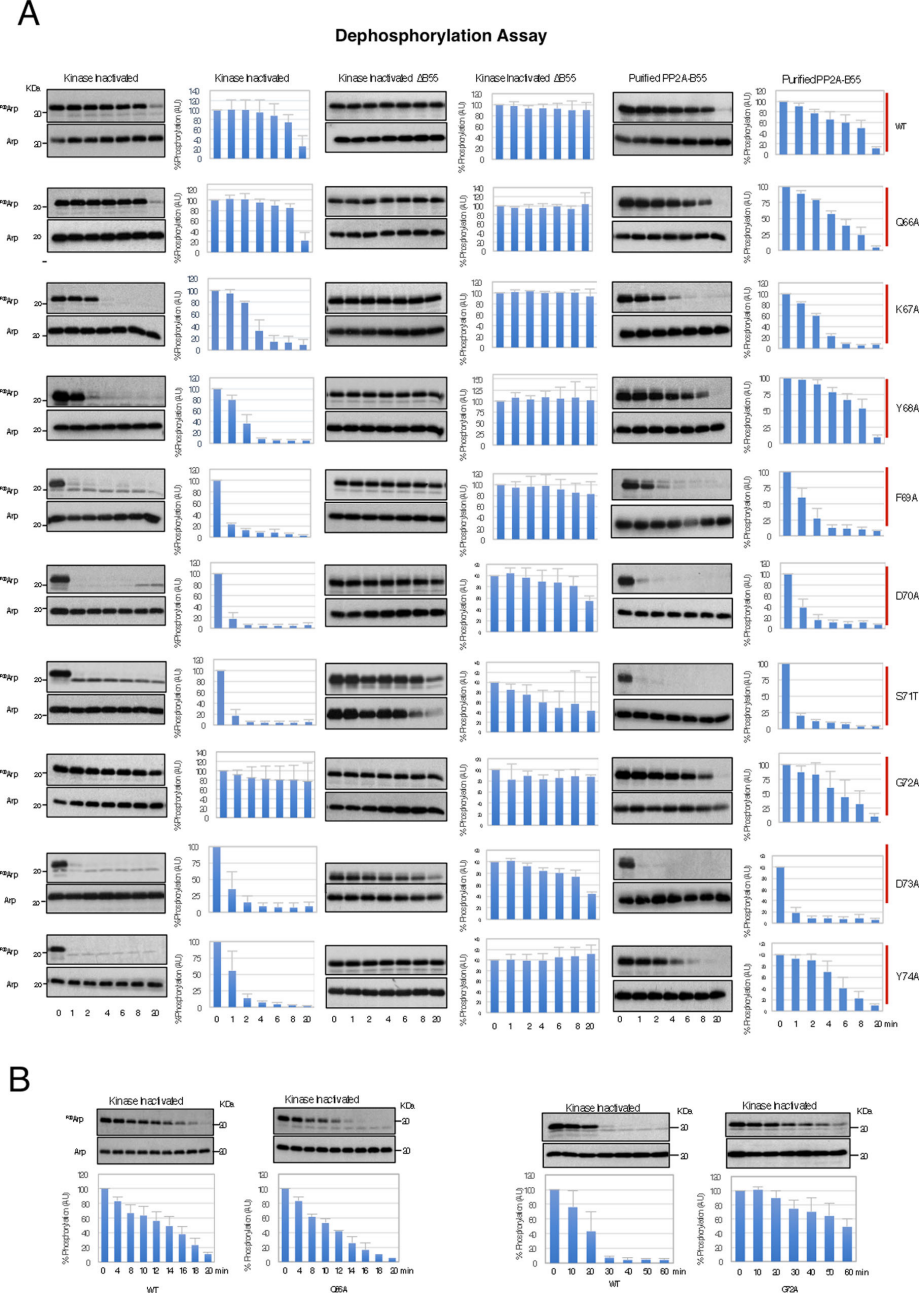
Aromatic and acidic residues flanking S67/S71 Gwl site are essential for slow Arpp19 dephosphorylation rate. **A** S67/S71 dephosphorylation of wild-type Arpp19 or of the indicated DSG Arpp19 mutants was measured in kinase-inactivated extracts depleted or not of B55, as well as upon the addition of a purified PP2A-B55 phosphatase by autoradiography (^P33^Arp). The amount of Arpp19 in each sample was assessed by western blot (Arp). The percentage of phosphorylation remaining with respect to the starting point calculated as in Fig. 1B and represented in bar graph as the mean value ± SD; *n* = 3 biological independent samples. **B** Dephosphorylation of the wild type and the indicated mutants of Arpp19 were performed in kinase-inactivated extracts and quantified in a bar graph as the mean value ± SD; *n* = 3 biological independent samples.

To test this hypothesis, we thus measured S67/S71 dephosphorylation of ‘in vitro’ phosphorylated mutants in kinase-inactivated egg extracts. The level of recombinant Arpp19 protein was evaluated by western blotting (Arp), whereas the rate of S67/S71 dephosphorylation was captured by autoradiography (^P33^Arp) (Fig. 2A, B). Interestingly, except for Q66A and G72A that kept normal or decreased dephosphorylation rates, respectively, all the other mutants of Arpp19 were rapidly (for K67A) or a very rapidly (for the rest) dephosphorylated on S67/S71. In agreement with Fig. 1B, this dephosphorylation pattern is promoted by PP2A-B55, as it was fully blocked by B55 depletion and is phenocopied by most mutants when ‘in vitro’ dephosphorylation assays were performed using a purified PP2A-B55. Y68A, Y74A and G72A are exceptions, with a dephosphorylation that was slightly faster in kinase-inactivated extracts (Fig. 2A). We do not know the reason for the differential behaviour of these mutants; however, it is possible that the higher concentration of C subunit present in purified PP2A-B55 could compensate the drop of Arpp19-PP2A C subunit interaction of these mutants resulting in a less-pronounced S67/S71 dephosphorylation effect.

**Fig. 3:**
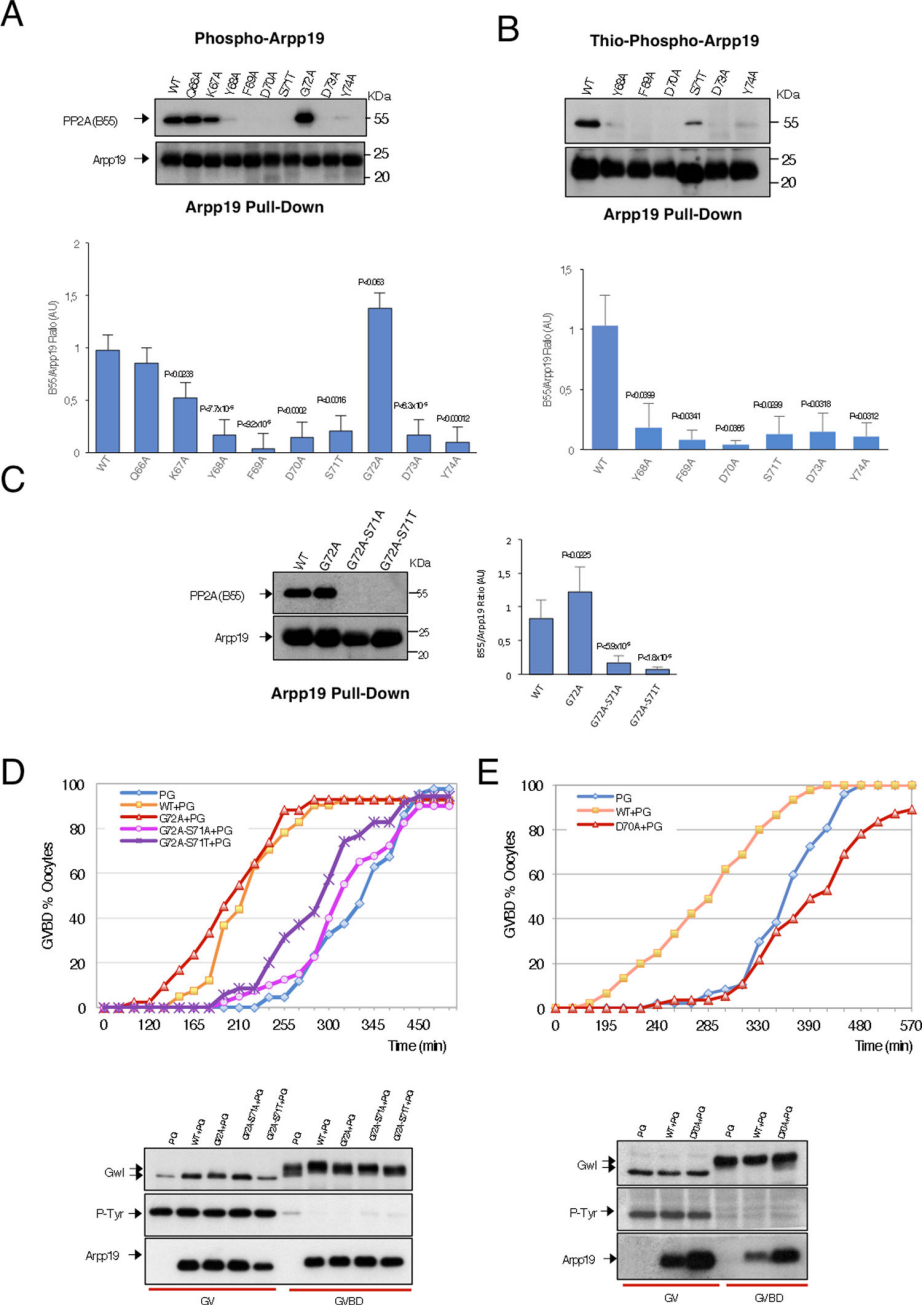
S67/S71 catalysis and PP2A-B55 interaction regulate each other to confer a proper timing of PP2A-B55 inhibition. **A** B55 levels associated to 20 ng of wild type or the indicated DSG mutants of His-Arpp19-pulldowns. Arpp19 amount in these pulldowns is also shown. Data were represented as mean B55/Arpp19 ratio ± SD. Two-tailed unpaired Student’s *t*-tests were performed in each pulldown to determine statistical relevance. *p* vs. wild-type Arpp19 is shown; *n* = 3 biological independent samples for mutants Y68A, G72A and Y74A; *n* = 5 for the D73A, *n* = 6 for the wild-type form and *n* = 4 for the rest. **B** The wild type and the indicated DSG mutant forms of Arpp19 were thio-phosphorylated and used for His-pulldown. B55 and Arpp19 levels were checked by western blotting and shown. The B55/Arpp19 ratios were quantified and represented in a bar graph as mean ± SD. Two-tailed unpaired Student *t*-tests were performed in each pulldown to determine statistical relevance. *p* vs. wild-type Arpp19 is shown; *n* = 3 biological independent samples. **C** The B55/Arpp19 ratios were obtained as in **A** for the indicated mutants and represented in a bar graph as mean ± SD. Two-tailed unpaired; *p* vs. wild-type Arpp19 is shown; *n* = 3 biological independent samples for the wild-type form; *n* = 8 for the G72A mutant, *n* = 5 for the G72A-S71A mutant and *n* = 3 for the G72A-S71T mutant. **D** Prophase oocytes were injected or not (PG) with 50 ng of the wild-type His-Arpp19 protein or with the indicated mutant forms and, 1 h later, treated with progesterone. GVBD was then scored as a function of time. Germinal vesicle (GV) and mature oocytes (GVBD) were western blotted to determine the levels of the injected protein, as well as the phosphorylation of Gwl and of the inhibitory site of Cdk1 Tyrosine 15. **E** As for **E**, except that the indicated mutant form of Arpp19 was used.

Interestingly, S67/S71 dephosphorylation rate correlated with the ability of these mutants to bind B55 (Fig. 3A) with a normal or an increased association for mutants displaying a regular or a slower dephosphorylation rate (Q66A and G72A, respectively), intermediate association for mutants with a moderate dephosphorylation (K67A) and with fully loss of B55 binding for mutants with a far faster dephosphorylation rate (Y68A, F69A, D70A, S71T, D73A, Y74A). The fastest dephosphorylation was observed when the acidic and aromatic residues of the DSG motif were mutated, the same mutants previously proposed to lose Gwl-dependent phosphorylation in human cells. Hence, our results confirm that the weak phosphorylation of these mutants at S67/S71 results from their dephosphorylation by PP2A-B55 and not from a decreased phosphorylation by Gwl^22^.

As expected, rapid dephosphorylation of S67/S71 and decreased binding to B55 in these mutants was also associated with their incapacity to promote mitotic entry upon their addition to Arpp19-depleted egg extracts (Supplementary Fig. 2A), indicating that although they still behave as PP2A-B55 substrates, they lost their phosphatase-inhibitory activity. Thus, loss of Arpp19 inhibitory activity is correlated with both S67/S71 phosphorylation and PP2A-B55 interaction; however, data above does not allow us to determinate whether S67/S71 dephosphorylation controls PP2A-B55 interaction or the opposite. Mutants could exclusively impact the catalysis of S67/S71 promoting a faster dephosphorylation of S67/S71 and resulting in a reduced residence time of Arpp19 on PP2A-B55, a process that would explain the loss of this interaction in our pulldown analysis. Conversely, changes in the local conformation of Arpp19 that would modify Arpp19-PP2A-B55 interaction could directly impact on S67/S71 catalysis increasing or reducing dephosphorylation and dissociation timings. To discriminate between these two possibilities, we blocked S67/S71 catalysis by phosphorylating ‘in vitro’, with ATPγS and recombinant Gwl kinase, DSG mutants displaying a rapid S67/S71 dephosphorylation and we monitored the association to PP2A-B55 as well as the inhibitory activity of these mutants (Fig. 3B and Supplementary Fig. 2B). When constitutively phosphorylated at S67/S71, none of these mutants restored their interaction to PP2A-B55 (Fig. 3B) or promote mitotic entry (Supplementary Fig. 2B), suggesting that these mutations might directly affect Arpp19-PP2A-B55 binding. This data supports the hypothesis of a control of S67/S71 dephosphorylation by Arpp19-PP2A-B55 interaction.

We next investigated the reverse hypothesis of whether S67/S71 catalysis could also modulate Arpp19 affinity to PP2A-B55. To this, we took advantage of the G72A mutant that displays an increased interaction to PP2A-B55 and we introduced an alanine or a threonine mutation at the S67/S71 site. Interestingly, G72A-S67/S71A and G72A-S67/S71T mutants failed to stably bind PP2A-B55, indicating that S67/S71 phosphorylation also modulates PP2A-B55 interaction (Fig. 3C). Together, the data above demonstrates that both S67/S71 catalysis and PP2A-B55 interaction regulate each other, to confer a proper timing of PP2A-B55 inhibition.

### Effect of DSG Arpp19 mutants in oocyte maturation

In order to investigate the impact of modifying Arpp19-PP2A-B55 affinity and S67/S71 dephosphorylation ‘in vivo’, we checked the effect on meiotic maturation upon PG treatment of the injection of prophase-arrested *Xenopus* oocytes with different Arpp19 mutants. We compared a wild-type Arpp19 form vs. either a G72A Arpp19 mutant with increased PP2A-B55 affinity, a non-phosphorylable G72A-S67/S71A or a rapid dephosphorylable G72A-S67/S71T double mutants, or a D70A Arpp19 mutant form with a fast dephosphorylation of S67/S71. The injection of wild-type Arpp19 results in an acceleration of germinal vesicle breakdown (GVBD) and meiotic resumption (Fig. 3D, PG vs. WT + PG). Interestingly, according with our data, the injection of G72A Arpp19 results in an acceleration of oocyte maturation compared to wild-type-injected oocytes (Fig. 3D, G72A + PG vs. WT + PG). However, this effect was fully lost when the G72A-S67/S71A or G72A-S67/71T double mutants were used. In these cases, oocytes followed a similar kinetics of maturation than non-injected PG-treated oocytes, indicating that only endogenous Arpp19 and not the double mutants displayed PP2A-B55 inhibitory activity. In the same line, oocytes injected with the D70A showed a similar maturation profile than non-injected oocytes treated with PG (Fig. 3E). Together, these ‘in vivo’ results confirm our ‘in vitro’ data supporting that Arpp19 phosphatase interaction and S67/S71 dephosphorylation control each other to define the correct PP2A-B55 inhibitory activity of this protein.

### Role of the C terminus of Arpp19 in PP2A-B55 inhibition

**Fig. 4:**
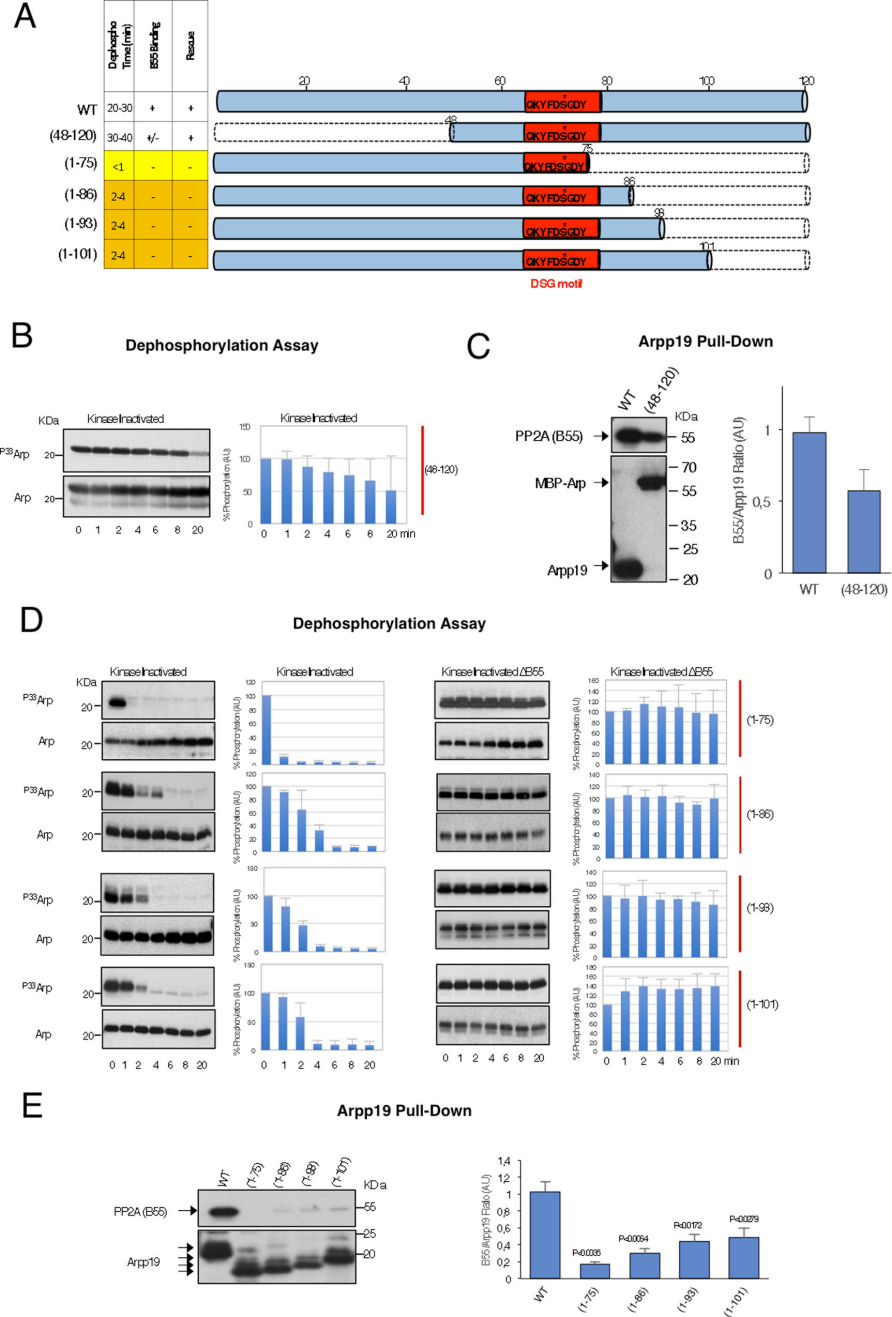
The C terminus of Arpp19 controls PP2A-B55 inhibition by modulating S67/S71 dephosphorylation rate. **A** Represented in dashed lines are the sequences deleted in the specified Arpp19 mutants. A table summarizing data of the S67/S71 dephosphorylation time in kinase-inactivated extracts as well as the binding or not to B55 and the capacity to restore the mitotic state in Arpp19-depleted extracts of all these mutants is also shown. Yellow and orange lines denote a dephosphorylation time of S67/S71 of <1 min or between 2 to 4 min, respectively. **B** The dephosphorylation of S67/S71 of (48–120) Arpp19 mutant was assayed in kinase-inactivated extracts and revealed by autoradiography. The amount of this Arpp19 mutant form in each sample is also shown. Bar graph shows mean percentage of phosphorylation remaining respect to the one of the starting point ± SD; *n* = 3 biological independent samples. **C** A volume of His-Arpp19 pulldown sample corresponding to 20 ng of wild type or (48–120) mutant is submitted to western blot and the associated B55 protein as well as the amount of Arpp19 present in the beads shown. Due to the insolubility of the His-Arpp19 (48–120) mutant, we used a double-tagged MBP-His-Arpp19. Quantification of the B55/Arpp19 ratio of three experiments was performed and represented as mean ratio ± SD; *n* = 3 biological independent samples. **D** S67/S71 dephosphorylation assays of the indicated Arpp19 mutants was tested in kinase-inactivated extracts depleted or not of B55 and revealed by autoradiography. The amounts of Arpp19 mutant proteins present in each sample was checked by western blotting. Results of three experiments are quantified and represented as the mean percentage of phosphorylation remaining ± SD; *n* = 3 biological independent samples. **E** Levels of B55 associated to a volume of beads equivalent to 20 ng of the wild type and the indicated C-terminal Arpp19 mutants. The amount of these mutants bound to the beads is also shown. The mean B55/Arpp19 and SD was calculated from three experiments and represented as a bar graph. Two-tailed unpaired Student’s *t*-tests were performed in each pulldown to determine statistical relevance. *p* vs. wild-type Arpp19 is shown; *n* = 5 biological independent samples for mutants (1–75) and (1–86), and *n* = 6 for the rest.

To ask whether sequences other than the DSG motif could be involved in the PP2A-B55 inhibitory activity of Arpp19, we mutated the N and the C terminus of this protein (Fig. 4A). N-terminal deletion mutant did not modify either S67/S71 dephosphorylation (Fig. 4B and Supplementary Fig. 1B) or its inhibitory capacity (Supplementary Fig. 3A) and only slightly decreased PP2A-B55 association (Fig. 4C). Conversely, the (1–75) Arpp19 mutant lacking most of the C-terminal region displayed a very fast dephosphorylation of the S67/S71 by PP2A-B55 (<1 min) (Fig. 4D). This dephosphorylation pattern was associated with a loss of its interaction with PP2A-B55 (Fig. 4E) and with its incapacity to promote mitotic progression in Arpp19-depleted egg extracts (Supplementary Fig. 3B). We thus constructed three longer C-terminal mutants (1–86, 1–93 and 1–101) (Fig. 4A). All these mutants also displayed an acceleration of PP2A-B55-dependent S67/S71 dephosphorylation (Fig. 4D) and a loss of the interaction with this phosphatase (Fig. 4E). Accordingly, they were unable to promote mitotic entry (Supplementary Fig. 3B). However, except for (1–75), these mutants rescued both mitotic entry capacity and binding to PP2A-B55 when S67/S71 was thio-phosphorylated (Supplementary Fig. 3C, D, respectively) indicating that the C terminus of Arpp19 regulates phosphatase-inhibitory capacity by modulating the dephosphorylation of S67/S71, with no major impact on the affinity of Arpp19 to PP2A-B55.

**Fig. 5:**
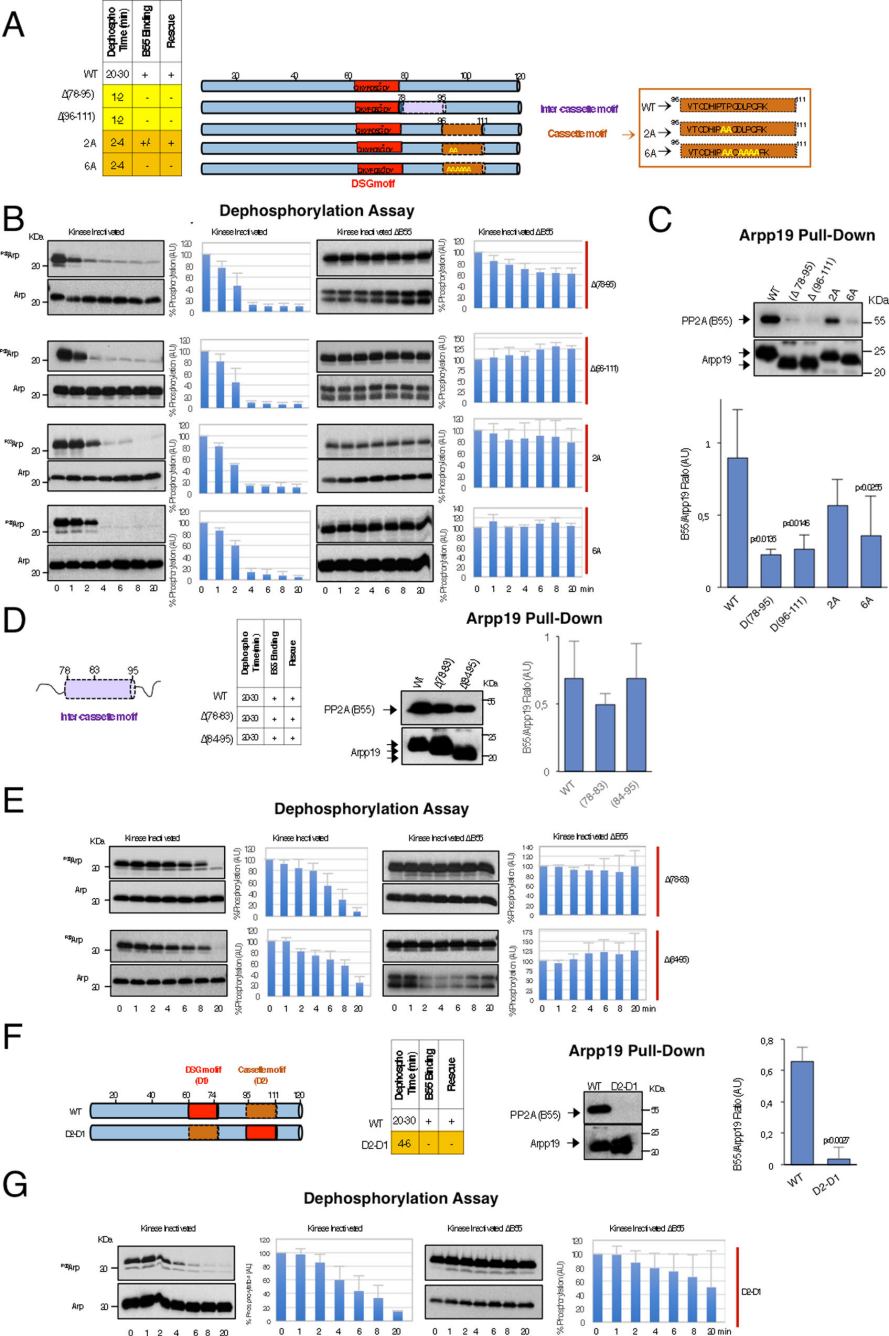
A specific sequence on the cassette motif of Arpp19 and a critical distance between this region and the DSG motif are essential for the correct timing of S67/S71 dephosphorylation. **A** Schematic representation of the ‘inter-cassette’ (sequence 78–95) and the ‘cassette’ (sequence 96–111) deleted regions of Arpp19, as well as the residues on the ‘cassette motif’ that have been mutated into alanine. **B** Dephosphorylation of S67/S71 of the indicated Arpp19 mutants in kinase-inactivated extracts devoid or not of B55. The amount of Arpp19 in each sample was assessed by western blotting. Data were quantified and represented as the mean percentage of phosphorylation remaining ± SD; *n* = 3 biological independent samples. **C** Western blotting showing the association of B55 to the indicated mutants of Arpp19 as well as the quantification of B55/Arpp19 mean ratio ± SD. Two-tailed unpaired Student’s *t*-tests were performed in each pulldown to determine statistical relevance. *p* vs. wild-type Arpp19 is shown; *n* = 3 biological independent samples for mutants D(78–95), D(96–111) and 2 A; *n* = 6 for the wild-type form and *n* = 4 for the 6A mutant. **D** Scheme depicting the two regions that have been deleted in the ‘inter-cassette’ motif. Table illustrating data on S67/S71 dephosphorylation timing as well as the capacity to bind B55 and to restore mitosis of the two mutants. Western blotting showing the amount of B55 present in the His-App19 pulldown assays of the indicated Arpp19 mutant forms. Graph bar representing the mean B55/Arpp19 ratio ± SD; *n* = 3 biological independent samples. **E** S67/S71 dephosphorylation assays of the indicated mutants of Arpp19 in kinase-inactivated extracts devoid or not of B55. Results were quantified and represented as the mean percentage of phosphorylation remaining ± SD; *n* = 3 biological independent samples. **F** A schematic of the DSG (D1) and the cassette (D2) regions of Arpp19 that have been exchanged in the D2-D1 mutant. A table with the dephosphorylation, binding and rescue results is also shown. The association of B55 to the D2-D1 mutant compared to the wild-type Arpp19 and the quantification of the mean B55/Arpp19 ± SD are shown. Two-tailed unpaired Student’s *t*-tests were performed in each pulldown to determine statistical relevance. *p* vs. wild-type Arpp19 is shown; *n* = 3 biological independent samples. **G** Dephosphorylation assay of S67/S71 of the wild type and the D2-D1 mutant in kinase-inactivated extracts that have been depleted or not of B55. Data from three experiments were represented as the mean percentage of phosphorylation remaining ± SD. *n* = 3 biological independent samples.

We next constructed shorter C-terminal mutants in which regions 78–95 or 96–11 were deleted (Fig. 5A). Deletion of each of these sequences promoted acceleration of PP2A-B55-dependent S67/S71 dephosphorylation (Fig. 5B), loss of the interaction with this phosphatase (Fig. 5C) and the inability to promote mitotic entry (Supplementary Fig. 3E). We next checked the conservation of these two sequences between α-endosulfine and among species. This analysis revealed a motif highly conserved at positions from 96 to 111 (Supplementary Fig. 3F), a motif that we named as the ‘cassette motif” (Fig. 5A). We thus constructed two different mutants in which two (T103A/P104A; 2A mutant) or six conserved aminoacids (T103A/P104A/D106A/L107A/P108A/Q109A; 6A mutant) were mutated into alanine. Interestingly, both 2A and 6A mutants (Fig. 5B) displayed an accelerated S67/S71 dephosphorylation. Moreover, PP2A-B55 binding was decreased for 2A and lost for 6A mutant (Fig. 5C). Finally, the 2A but not 6A mutant conserved its ability to trigger mitosis (Supplementary Fig. 4A). However, this faculty, as well as its capacity to bind PP2A-B55, were rescued in the latter mutant when S67/S71 was thio-phosphorylated (Supplementary Figs. 4B and 3D, respectively), hence confirming that the C-terminal cassette motif of Arpp19 is essential to confer correct dephosphorylation catalysis of the Gwl site. Moreover, it demonstrates that this capacity is sequence specific.

Unlike the cassette motif, the sequence from 78 to 95 (called hereafter as the ‘inter-cassette motif’) (Fig. 5A, D) appears to be less conserved (Supplementary Fig. 3F); yet, we decided to test whether the length instead of the sequence identity of this region would be important for Arpp19 to preserve its PP2A-B55 binding and inhibitory activity. We thus constructed two mutants in which either region 78–83 or region 84–95 were deleted (Fig. 5D). Both of these deletions promoted a standard interaction with PP2A-B55 (Fig. 5D), a normal dephosphorylation of S67/S71 (Fig. 5E) and mitotic entry in Arpp19-depleted egg extracts (Supplementary Fig. 4C).

This indicates that no specific sequence in these zones is required but instead a minimal length of the inter-cassette motif is critical. Thus, a distance between the DSG and the cassette motifs determines the correct inhibitory activity of Arpp19.

From this data, we conclude that the presence of two sequence-specific regions is essential for Arpp19 inhibitory activity, the DSG (D1) and the cassette (D2) motifs (Fig. 5F). As Arpp19 is an intrinsically disordered protein, we next wondered whether, beside sequence specificity and length, the localization of these two regions in the protein was also important for their physiological function. We thus constructed a chimeric Arpp19 protein in which these two regions were exchanged (Fig. 5F). Interestingly, although this chimeric protein was normally phosphorylated in S67/S71, this residue was fastly dephosphorylated (Fig. 5G) and fully unable to bind PP2A-B55 (Fig. 5F) or to promote mitosis (Supplementary Fig. 4D) even upon thio-phosphorylation of S67/S71 (Supplementary Figs. 3D and 4E). Hence, as for aromatic and acidic DSG mutants, the location of these two regions into the protein is critical to maintain PP2A-B55-Arpp19 stable interaction and to prevent S67/S71 rapid catalysis and loss of phosphatase-inhibitory activity.

### S109/S113 phosphorylation modifies S67/S71 dephosphorylation

**Fig. 6:**
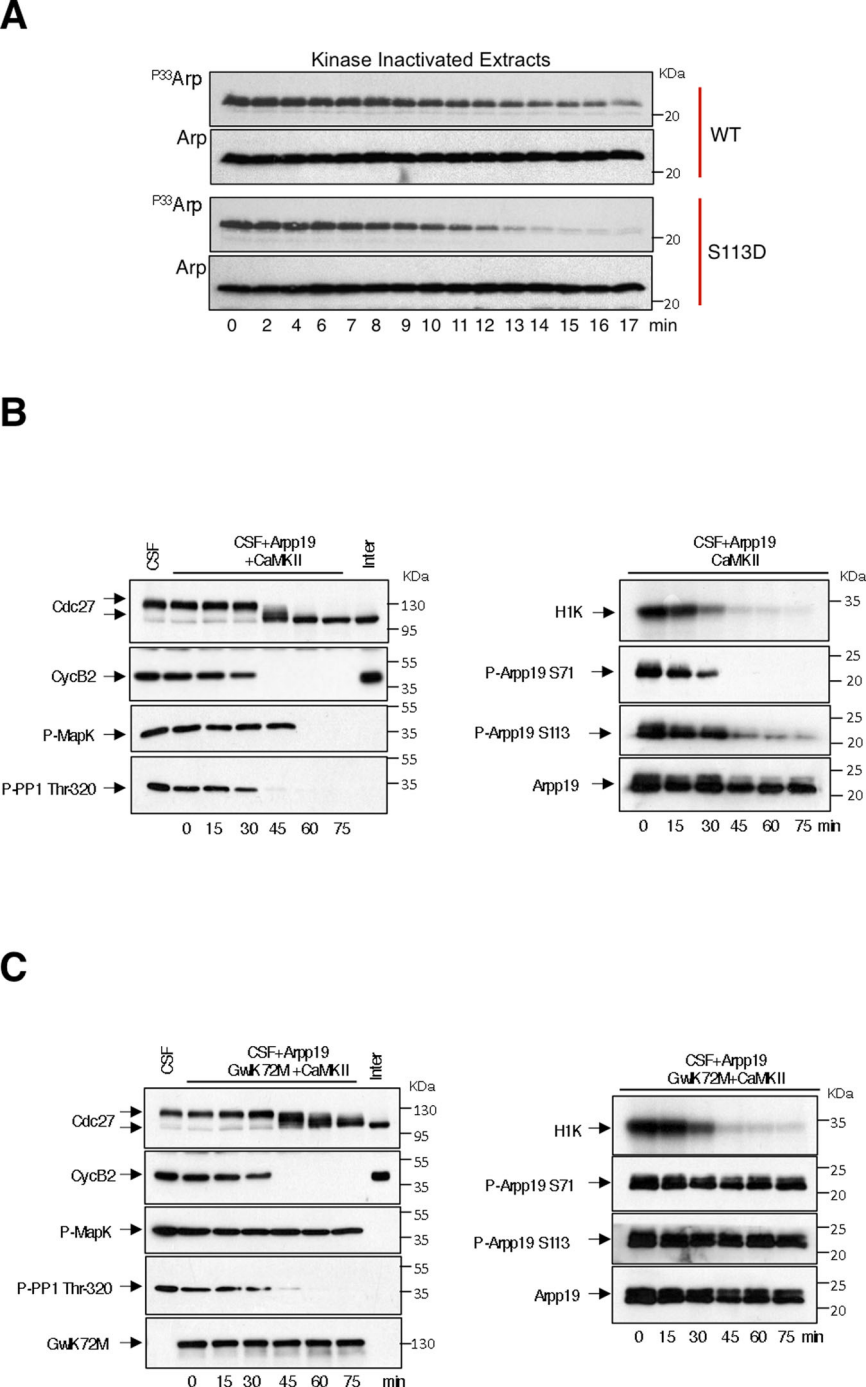
Phosphorylation of S109/S113 modifies the temporal pattern of S67/S71 dephosphorylation. **A** Wild type and S109/S113D Arpp19 mutant were phosphorylated ‘in vitro’ with of [γ^33^P] ATP by GwlK72M on S67/S71 and supplemented to kinase-inactivated *Xenopus* egg extracts. The dephosphorylation of this residue was analysed at the indicated times by autoradiography (^P33^Arp) and the amount of Arpp19 in each sample measured by western blotting (Arp). **B** CytoStatic Factor (CSF) egg extracts were supplemented with a trace level of Arpp19-purified protein and activated to exit meiosis by the addition of active CamKII. The levels and dephosphorylation of the indicated proteins were analysed by western blotting, whereas Cyclin B/Cdk1 activity was measured by histone H1 phosphorylation (H1K). ‘Inter’ denotes interphase egg extracts. **C** As for **C**, except that S67/S71 Arpp19 dephosphorylation and PP2A-B55 reactivation upon meiosis exit in these extracts was blocked by the concomitant addition of GwlK72M-purified protein.

Our results indicate that the cassette motif is essential for Arpp19 physiological activity by modulating the catalysis of PP2A-B55-dependent dephosphorylation of S67/S71. Interestingly, S109/S113, close to this motif, is phosphorylated by PKA in prophase *Xenopus* oocytes and this phosphorylation is essential to block meiotic resumption^18^. Due to the close proximity, we hypothesized that S109/S113 phosphorylation could modulate the cassette motif and, thus, have an impact on S67/S71 dephosphorylation and PP2A-B55 inhibitory activity. Accordingly, S67/S71 dephosphorylation of Arpp19 was significantly faster in S109/S113D Arpp19 mutant compared to wild-type Arpp19 (Fig. 6A), suggesting that S109/S113 phosphorylation is a key site that modulates PP2A-B55 inhibitory activity of Arpp19.

Although PKA has been characterized as the kinase promoting the phosphorylation of this residue^17,18^, nothing is known about the counterbalancing phosphatase. We thus, focused on identifying this phosphatase.

To this end, we checked S109/S113 and S67/S71 dephosphorylation using a trace amount of Arpp19 in CytoStatic Factor (CSF) extracts. These extracts were then forced to exit meiosis by the addition of active CamKII. Both S67/S71 and S109/S113 were fully dephosphorylated at meiotic exit, although S67/S71 dephosphorylation was significantly faster than S109/S113 (Fig. 6B). Interestingly, when S67/S71 phosphorylation was maintained by adding a Gwl hyperactive form, cyclin B degradation and CSF exit was normally performed (Fig. 6C). However, S109/S113 dephosphorylation was not observed strongly, suggesting that this dephosphorylation depends on PP2A-B55 activity.

### S109/S113 Arpp19 dephosphorylation depends on phospho-S67/S71

**Fig. 7:**
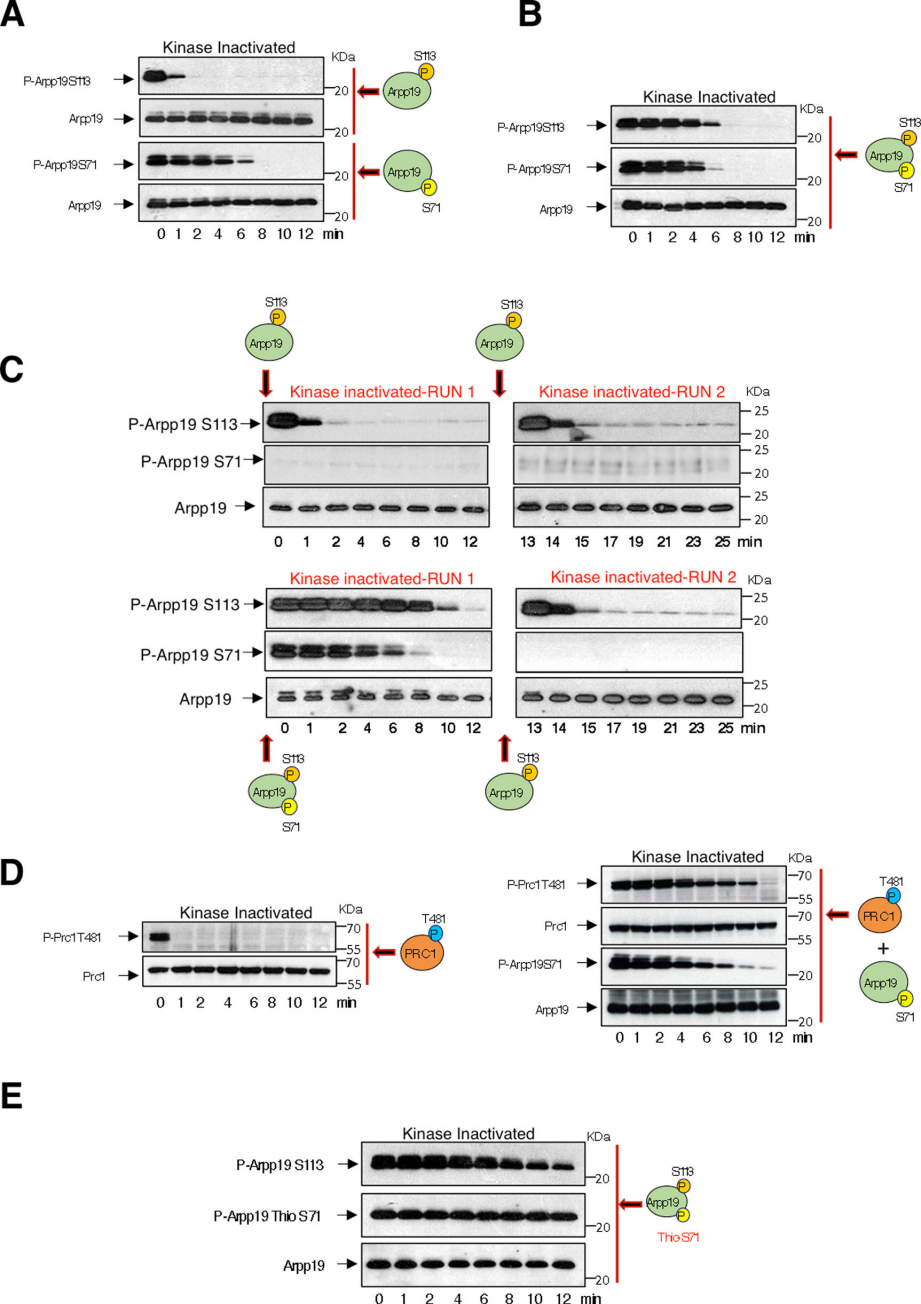
Dependency of S109/S113 dephosphorylation on the phosphorylation of S67/S71 of Arpp19. **A** Arpp19 phosphorylated ‘in vitro’ in S67/S71 or S109/S113 residues by Gwl or PKA, respectively, were separately supplemented to kinase-inactivated *Xenopus* egg extracts and the dephosphorylation rate of each site, as well as the total amount of this protein were analysed by western blotting. **B** Arpp19 ‘in vitro’-phosphorylated by Gwl and Arpp19 ‘in vitro’-phosphorylated by PKA were mixed together into kinase-inactivated extracts and the dephosphorylation of each of these sites analysed at the indicated time points. **C** In a first run of dephosphorylation, a pulse of Arpp19 phosphorylated by PKA or by both PKA and Gwl was supplemented to kinase-inactivated extracts. Samples were then recovered at the indicated time points. After 12 min, a second round of dephosphorylation was performed in these extracts upon the re-addition of a new pulse of Arpp19 phosphorylated ‘in vitro’ by PKA on S109/S113. Phosphorylation of S67/S71 and S109/S113 in the samples were evaluated by western blotting with specific phospho-antibodies. **D** Prc1 was phosphorylated ‘in vitro’ by purified Cyclin A/Cdk and supplemented alone (left panels) or together with phospho-S67/S71 Arpp19 (right panels) to kinase-inactivated extracts and the phosphorylation of T481 of Prc1 and S67/S71 of Arpp19, as well as the amount of these two proteins were examined by western blotting. **E** Arpp19 phosphorylated ‘in vitro’ by PKA on S109/S113 and thio-phosphorylated on S67/S71 by GwlK72M were mixed into kinase-inactivated extracts. Dephosphorylation of these two residues were then measured at the indicated time points by western blotting. Data shown in the figure are representative of at least three different experiments.

To further elucidate whether PP2A-B55 is responsible of S109/S113 dephosphorylation, we used kinase-inactivated extracts supplemented with either a phospho-S109/S113 or a phospho-S67/S71 Arpp19 protein. As expected from the absence of kinase activity in these extracts, dephosphorylation of both S109/S113 and S67/S71 of Arpp19 was drastically accelerated (2–6 min) (Fig. 7A) when compared to the one observed in CamKII-treated CSF extracts (30–45 min) (Fig. 6C). Moreover, S109/S113 was dephosphorylated before S67/S71 (Fig. 7A). Interestingly, this inversed order of dephosphorylation was no longer observed when extracts were simultaneously supplemented with both S67/S71 and S109/S113 phosphorylated Arpp19 forms (Fig. 7B). These results indicate that S67/S71 site modulates S109/S113 dephosphorylation.

We next used two kinase-inactivated extract samples. In one of these samples, phospho-S109/S113 Arpp19 was added, whereas the other one was supplemented with both phospho-S67/S71 and phospho-S109/S113 Arpp19 forms. Dephosphorylation of S109/S113 and S67/S71 was then followed during 12 min (Fig. 7C, RUN 1). After this period of time, both extract samples were supplemented again with phospho-S109/S113 Arpp19 and the dephosphorylation of this residue was recorded for an additional period of 12 min (Fig. 7C, RUN 2). Interestingly, dephosphorylation of S109/S113 was very fast (about 1–2 min) in both runs in the extract sample where only phospho-S109/S113 Arpp19 was supplemented. Conversely, during the first run, a dependence of dephosphorylation of S109/S113 on S67/S71 dephosphorylation was observed in the extract sample in which phospho-S67/S71 and phospo-S109/S113 were simultaneously added. However, in this sample, S109/S113 dephosphorylation acquired again a fast dephosphorylation rate during the second run when phospho-S67/S71 Arpp19 was absent. This data confirms a regulatory role of S67/S71 phosphorylation on S109/S113 dephosphorylation.

Phospho-S67/S71 Arpp19 is known to be a highly competitive substrate of PP2A-B55 that negatively regulates this enzyme^16^. It is thus possible that as for S67/S71, dephosphorylation of S109/S113 could be catalysed by PP2A-B55 and, consequently, be slowed down when Arpp19 is phosphorylated at S67/S71. To test this hypothesis, we first compared the dephosphorylation pattern of another well-known substrate of PP2A-B55, phospho-T481 Prc1. T481 Prc1 dephosphorylation is very rapid in the absence of phospho-S67/S71 Arpp19 (Fig. 7D). However, it was maintained until S67/S71 Arpp19 was fully dephosphorylated (Fig. 7D). Finally, we assessed the impact on S109/S113 dephosphorylation of a thio-phosphorylated S67/S71 Arpp19. As expected, phospho-S109/S113 was mostly stable under these conditions (Fig. 7E), indicating that Arpp19 dephosphorylation at S109/S113 mostly relies on PP2A-B55.

### PP2A-B55 dephosphorylates S109/S113 of Arpp19

**Fig. 8:**
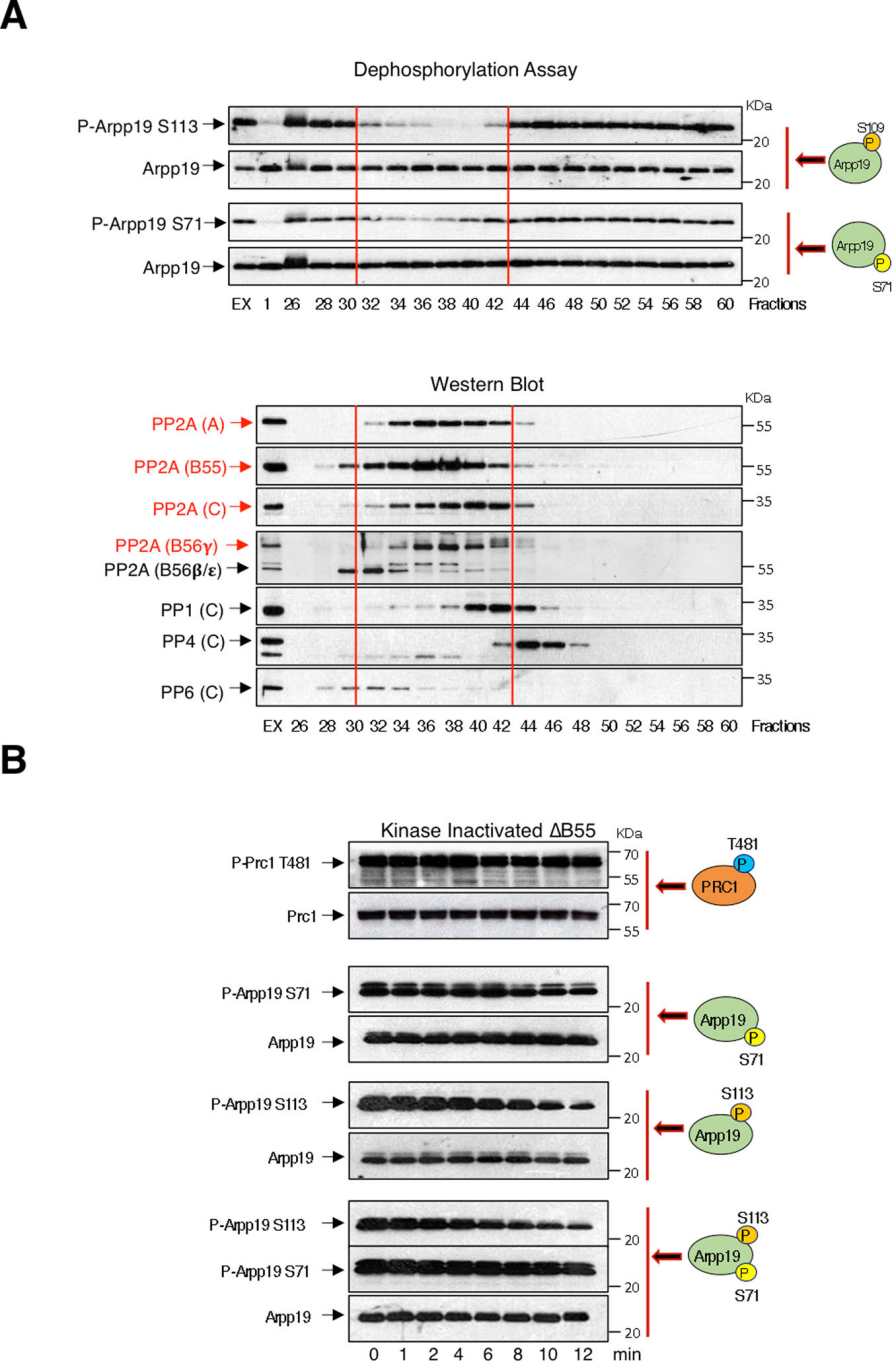
PP2A-B55 is the phosphatase responsible of the dephosphorylation of Arpp19 on S109/S113. **A** Kinase-inactivated *Xenopus* egg extracts were submitted to gel filtration chromatography and eluted fractions subsequently supplemented with Arpp19 phosphorylated ‘in vitro’ on either S109/S113 or S67/S71. The dephosphorylation of these residues and the amount of Arpp19 were then measured by western blotting (upper panels, dephosphorylation assay). ‘EX’ and 1 correspond to phosphorylation at time 0 and 10 minutes respectively, of the indicated residues upon directly mixing with a kinase-inactivated extract. The presence of the indicated proteins in elution fractions and in the kinase-inactivated extract sample were assessed by western blotting (lower panels, western blotting). Red lines highlight the fractions displaying S109/S113 and S67/S71 dephosphorylation activity. The name of the proteins whose level picked in these fractions are also depicted in red. **B** Prc1 ‘in vitro’ phosphorylated on T481 by purified Cyclin A/Cdk and Arpp19 phosphorylated on either S67/S71 or on S109/S113 were supplemented together or separately to kinase-inactivated extracts depleted of B55 and the dephosphorylation of the corresponding residues analysed over the time by western blotting. Data of the figure are confirmed in three different experiments.

To fully elucidate the identity of the S109/S113 Arpp19 phosphatase, we performed a biochemical approach in which kinase-inactivated extracts were fractionated by gel chromatography using a Superdex 200 column. The different fractions were then tested for S109/S113 Arpp19 dephosphorylation activity using recombinant Arpp19 phosphorylated ‘in vitro’ on either S109/S113 or on S67/S71 as a substrate. S109/S113 and S67/S71 dephosphorylation activities were both recovered at fractions 32–42 overlapping with the peak of PP2A-A, C, B55 and B56 abundance, supporting again the hypothesis of PP2A being responsible for these dephosphorylations (Fig. 8A). Although both B55 and B56 were present in S109/S113 dephosphorylation-active fractions, we decided to focus on the putative involvement of PP2A-B55, given our results obtained in extracts using S67/S71 thio-phosphorylated Arpp19. We first monitored the stability of S109/S113 phospho-site in B55 immunodepleted interphase extracts. As expected, both T481 of Prc1 and S67/S71 of Arpp19, two known substrates of PP2A-B55, were not dephosphorylated in B55-depleted interphase extracts (Fig. 8B). Similarly, S109/S113 dephosphorylation was dramatically delayed in kinase-inactivated extracts devoid of B55, regardless of the presence of a S67/S71-phosphorylated Arpp19 form, indicating again that PP2A-B55 is the main phosphatase involved in dephosphorylation of S109/S113 of Arpp19.

**Fig. 9:**
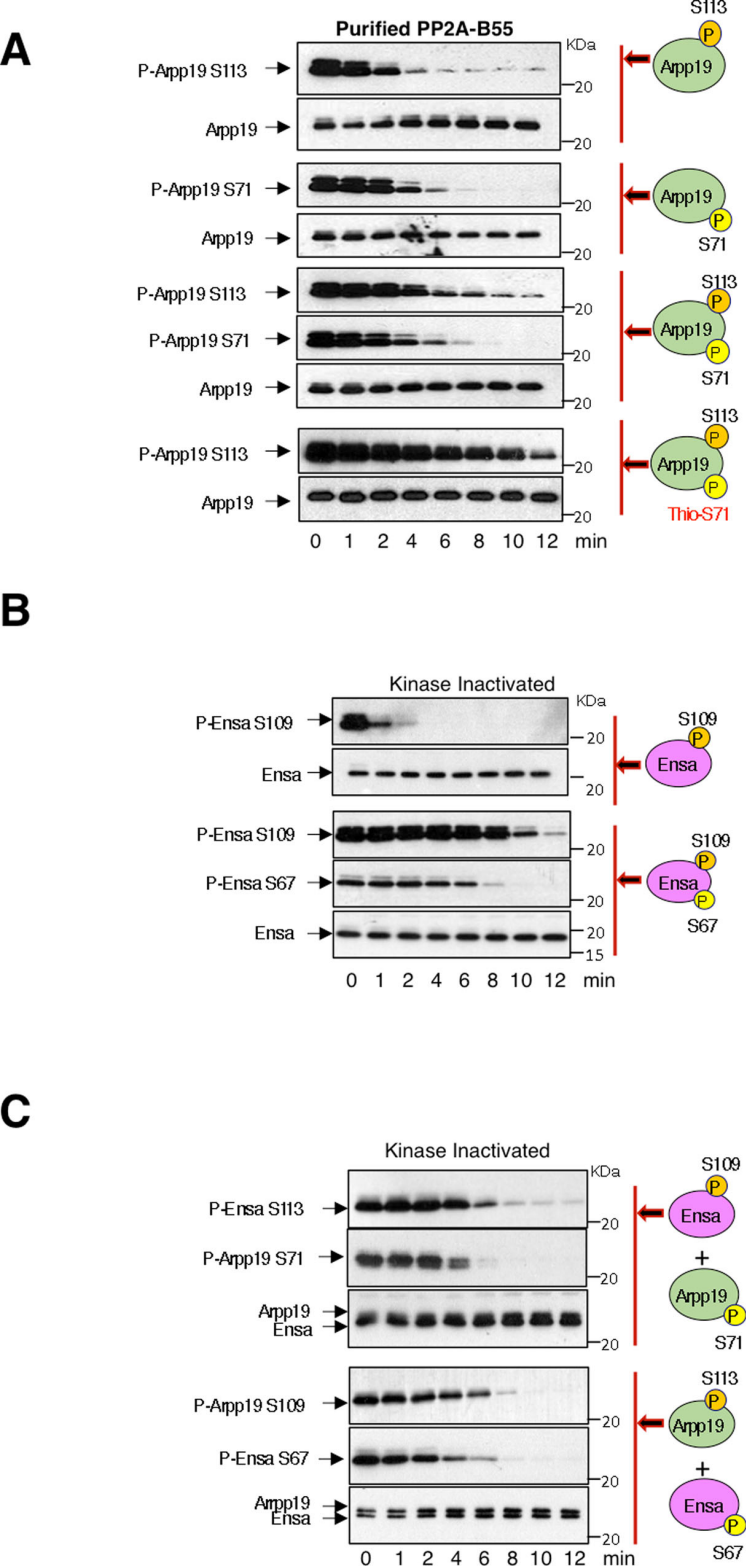
Phospho-S67/S71 negatively regulates dephosphorylation of S109/S113 of Arpp19 by PP2A-B55. **A** Arpp19 was phosphorylated ‘in vitro’ on S109/S113, S67/S71 or thio-phosphorylated on S67/S71, supplemented alone or with the indicated combinations to kinase-inactivated extracts and the temporal pattern of dephosphorylation analysed. **B** ENSA protein was phosphorylated ‘in vitro’ on S67 by GwlK72M or S109 by PKA and supplemented alone or combined as indicated to kinase-inactivated extracts and the dephosphorylation time of these residues, as well as the amount of ENSA protein examined. **C** Phospho-S109 ENSA and phospho-S67/S71 Arpp19 or phospho-S109/S113 Arpp19 and phospho-S67 ENSA were simultaneously added into kinase-inactivated extracts and the timing of dephosphorylation of the different phospho-sites analysed. Experiments supporting the data of this figure were performed at least three times.

We subsequently confirmed this data ‘in vitro’ using a purified PP2A-B55 phosphatase (Supplementary Fig. 5A and Fig. 9A). We assessed the dephosphorylation of the S109/S113 and S67/S71-phosphorylated forms of Arpp19 when separately or simultaneously added to the reaction mix. In agreement with our data above, the temporal pattern of S67/S71 dephosphorylation was unchanged under all conditions. On the contrary, S109/S113 phosphorylation disappeared very rapidly in the absence of phospho-S67/S71 but did not occurred until full S67/S71 dephosphorylation when this phospho-residue was present. Finally, we checked the effect of the double addition to the reaction mix of a phospho-S109/S113 and a thio-phospho-S67/S71 Arpp19 form. Under these conditions, S109/S113 phosphorylation was stabilized throughout the experiment confirming that PP2A-B55 is the main phosphatase involved in S109/S113 dephosphorylation. Interestingly, this regulatory mechanism is shared and interchangeable by the other PP2A-B55 inhibitor ENSA (Fig. 9B, C).

## Discussion

Although the essential function of Arpp19-dependent PP2A-B55 inhibition in mitosis and meiosis has been well established, little is known about the molecular mechanisms controlling this inhibition. It is known that ENSA acts as a highly competitive substrate of this phosphatase with a tight-binding to this enzyme and a slow dephosphorylation rate, thus reducing PP2A-B55 substrate dephosphorylation by competition^16^. This competition model implies a dependence of the inhibitory capacity of these proteins on two main parameters as follows: (1) the ability of Arpp19/ENSA to bind PP2A-B55, reinforced by its Gwl-dependent phosphorylation on S67/S71 and (2) the subsequent capacity of these proteins to dissociate from PP2A-B55 via the dephosphorylation of this residue. It is therefore essential to elucidate the structural properties defining phosphatase interaction and S67/S71 dephosphorylation rate to understand the molecular mechanisms of Arpp19-dependent PP2A-B55 inhibition.

Here we performed an extended structure/function study to determine these properties.

We first tested the involvement of the KKR residues flanking the DSG motif of Arpp19 in PP2A-B55 binding. Our data suggest that KKR residues would reduce Arpp19-PP2A-B55 interaction.

**Fig. 10:**
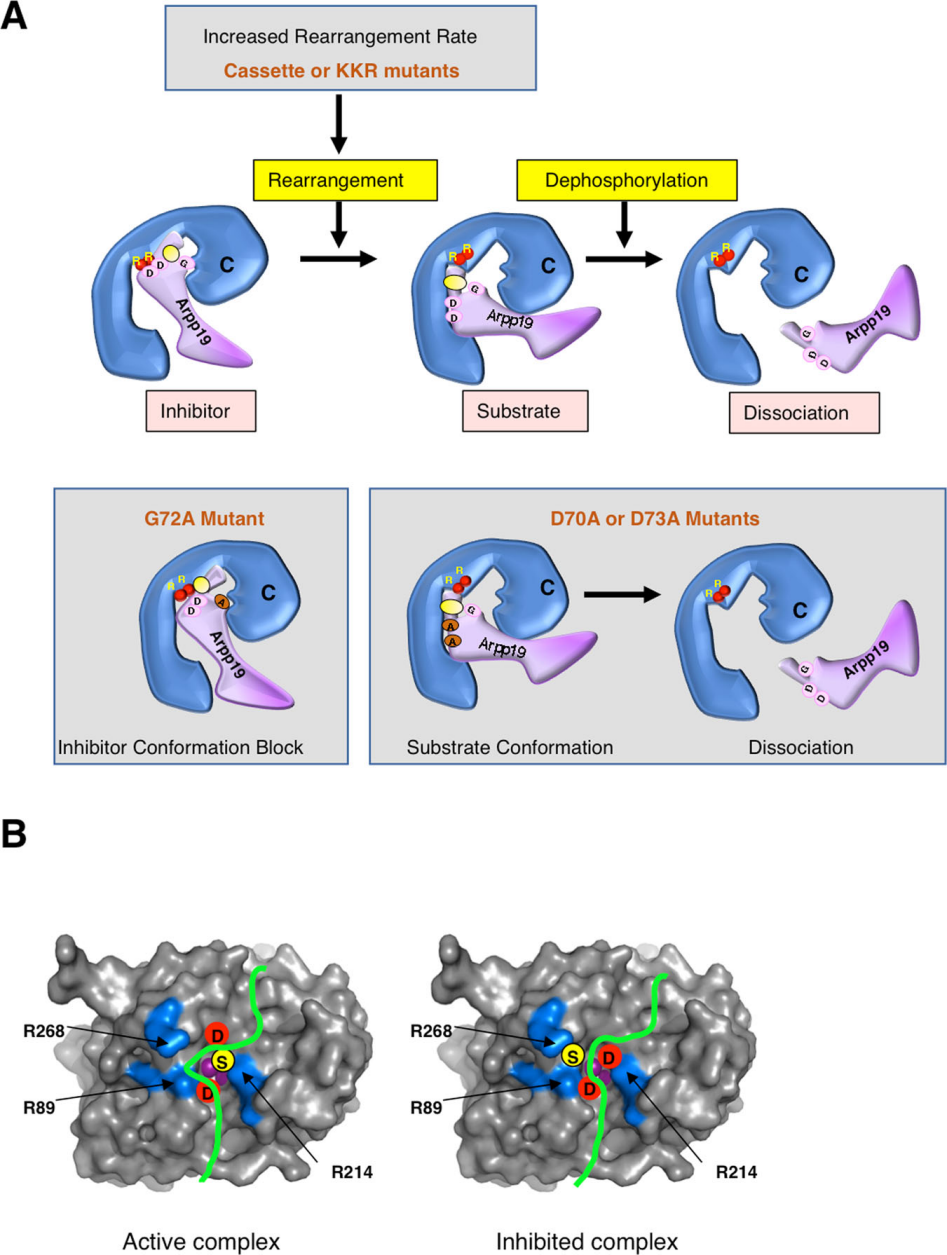
Putative docking of the DSG motif of Arpp19 into the active site of PP2A-B55. **A** The docking of phospho-S67/S71 of Arpp19 into PP2A catalytic subunit (C) as an inhibitor conformation, its rearrangement towards a substrate conformation and its final dissociation upon dephosphorylation. Phosphorylation of S67/S71 is represented (yellow circle). The two aspartic residues D70 and D73 (pink circles) that could interact with the metallic centre (red circles) and with the two arginine residues (R) of the PP2A C subunit are shown. The putative effects of KKR/AAA, G72A, and D70A/D73A mutants of Arpp19 on the docking conformation of phospho-S67/S71 are indicated. **B** Putative docking of the DSG motif of Arpp19 into the active site of PP2A-B55 (PDB2IE4)^24^. Left: conformation of the peptide orientating the phosphorylated serine (yellow circle) toward the catalytic centre with the two aspartates (red circles) pointing toward nearby arginines (R89 and R214). Right: inhibited form in which the phosphorylated serine points outside (toward R268), while the dications (violet spheres) from the catalytic centre are chelated by one or two aspartates from the DSG motif.

We next investigated the role of the DSG motif and of the C-terminal region of Arpp19 in phosphatase-inhibitory activity. Despite these two mutant types promoting similar phenotypes, namely decreased Arpp19-PP2A-B55 interaction, rapid S67/S71 dephosphorylation and loss of inhibitory activity, our data support a different contribution of these regions in the modulation of PP2A-B55 activity. Accordingly, phenotypes induced by DSG mutants are not affected by S67/S71 thio-phosphorylation, indicating that their effect is independent of S67/S71 catalysis. Conversely, deletions and mutations of the C terminus of Arpp19 are mostly rescued by thio-phospho-S67/S71, highlighting a main effect of these mutants on the modulation of the catalysis of this site. To explain these results, we propose a model in which phospho-S67/S71 would bind PP2A-B55 catalytic site in a competitive constrained conformation (Fig. 10A, Inhibitor). This conformation would be propitious for stable interaction but not for dephosphorylation and would slowly be rearranged to favourably orient the leaving phosphate group (Fig. 10A, Substrate). Our data support the hypothesis of an essential role of the DSG residues on the correct docking of the phospho-S67/S71 in a competitive constrained conformation. On the contrary, the C-terminal regions would be mostly controlling rearrangement of the inhibitory conformation of phospho-S67/S71 Arpp19 towards a substrate conformation and thus controlling its catalysis rate (Fig. 10A). In this model, a decreased interaction due to an improper docking of the phosphate of S67/S71 on the catalytic site would prevent its precise positioning as a competitive constrained conformation, and would result in a rapid dephosphorylation of this site and in a loss of its inhibitory activity. Conversely, an increased S67/S71 dephosphorylation rate promoted by a rapid inhibitory/substrate conformation rearrangement would result in its rapid dissociation from the phosphatase and a similar loss of its inhibitory activity. Accordingly, our data indicate that PP2A-B55-Arpp19 binding and S67/S71 dephosphorylation regulate each other to confer a correct temporal pattern of phosphatase inhibition.

The strict conservation of the DSG motif does not allow the precise prediction of the putative structural role of each of the residues mutated in this study but suggests its important role in adopting a resting conformation of Arpp19 as a competitive slow substrate. Considering previously reported PP2A C subunit structural data^24^, we hypothesize that the flexibility of G72 residue would participate to the facile exchange between the two conformations and, consequently, mutations on this site would block phospho-S67/S71 in its inhibitory conformation. This hypothesis is supported by our ‘in vitro’ data on G72A Arpp19 mutant displaying increased interaction to PP2A-B55 and decreased dephosphorylation of S67/S71 and our ‘in vivo’ data showing an acceleration of meiotic resumption likely induced by an enhanced inhibitory activity of this mutant. The large aromatic side chains of Y68, F69, and Y74 could also favour tight interactions with the phosphatase in the competitive conformation. This may involve cross-talks or synergy between these vicinal and hydrophobic aminoacids. The two aspartate residues, D70 and D73, may similarly regulate local conformation and precise interactions with the phosphatase but an additional role in the observed inhibition could come into play. Indeed, they could also prevent the proper orientation of the phosphorylated S67/S71 toward the catalytic centre by competing with this residue for the direct interaction with the metallic centre of the phosphatase and/or with the two arginines of the PP2A C subunit pointing into the catalytic site (R89 and R214 of human PP2A C subunit) (Fig. 10B)^24^. Interestingly, this internal competition of D70/D73 with the phosphate of S67/S71 to bind the key catalytic residues of PP2A would not be present in the S67/S71T mutation favouring the rapid dephosphorylation of this mutant. Indeed, the mutation into alanine of these aromatic and negatively charged residues of the DSG transformed this inhibitor into a regular PP2A-B55 substrate because of the loss capacity to be docked in its particular and precise inhibitory conformation. We observed an accelerated rate of S67/S71 dephosphorylation, thereby conferring to this residue a similar temporal pattern of dephosphorylation than other PP2A-B55 substrates such as PRC1. Interestingly, as expected from this hypothesis, the double mutant G72A-D70A turned out to be a rapid substrate of PP2A-B55 (Supplementary Fig. 5B), suggesting that when the inhibitory conformation cannot be achieved, the mutation of G72 likely involved in inhibitory/substrate conformation rearrangements does not affect rapid S67/S71 dephosphorylation. Similar, the internal competition of D70 and D73 with phosphorylated S67/S71 for binding to the key catalytic residues of PP2A would not be present in the S67/S71T mutant. The threonine might not be accommodated in the inhibitory conformation, and this would in turn favours its rapid dephosphorylation.

The distant sites such as the KKR region and the cassette motif could contribute to the optimal inhibitory/substrate rearrangement rate of phospho-S67/S71 upon their docking onto the phosphatase surface. Moreover, a minimal distance is required between this region and the DSG motif for a correct inhibitory pattern.

Besides S67/S71, a phosphorylation of Arpp19 on S109/S113, has also been identified^17,18^. Notably, it has been shown that this residue is maintained phosphorylated by PKA in prophase I-arrested oocytes and partially drops upon PKA inhibition induced by PG^18^. Our data show that phospho-S109/S113 modulates S67/S71 dephosphorylation, a regulation that could play an essential role in the establishment of the correct temporal pattern of PP2A-B55 inhibition and Cyclin B/Cdk1 activation during oocyte maturation. We also identified PP2A-B55 as the phosphatase responsible of the dephosphorylation of this residue. Moreover, we demonstrate that its dephosphorylation depends on the previous dephosphorylation of S67/S71, highlighting a double feedback loop by which these two residues control each other’s PP2A-B55-mediated dephosphorylation. This feedback loop would play an essential role in the maintenance of the correct balance of these two phosphorylations conferring the correct temporal regulation of PP2A-B55 and Cyclin B/Cdk1 activation during oocyte maturation and meiosis exit.

In summary, this study established the main structural basis of controlling Arpp19 S67/S71 dephosphorylation and PP2A-B55 inhibitory activity. In addition, we identified the regulatory mechanism by which S109/S113 modulates this phospho-site. We finally discovered a double feedback loop between these two sites that would coordinate the proper temporal pattern of Arpp19-dependent PP2A-B55 inhibition and Cyclin B/Cdk1 activation during meiotic progression and exit. Further studies will be required to determine whether this tuned regulation of Arpp19 also takes place during mitosis to timely ensure the accurate entry and progression.

## Methods

### *Xenopus laevis* induction and husbandry

Regulation for the use of *Xenopus laevis*, as outlined in the Animal Scientific Procedures Act, and implemented by the Direction Générale de la Recherche et Innovation, Ministère de l’Enseignement Supérieur de l’Innovation of France, were followed. Frogs were obtained from Centre de Ressources Biologiques Xénopes (CRB) of Rennes, France, and kept in a *Xenopus* research facility at the CRBM (Facility Centre approved by the French Government. Approval number B34-172-39). Females were injected with 500 U Chorulon (Human Chorionic Gonadotrophin) and 18 h later laid oocytes were used for experiments. Adult females were exclusively used to obtain eggs. All procedures were approved by the Direction Generale de la Recherche et Innovation, Ministère de L’Enseignement Supérieur de la l’Innovation of France (Approval number APAFIS#4971-2016041415177715v4).

### *Xenopus* oocytes and egg extracts

Prophase I-arrested oocytes were obtained after surgery, washed in MMR buffer (25 mM NaCl, 0.5 mM KCl, 0.25 MgCl_2_, 0.025 mM NaEGTA, 1.25 mM HEPES-NaOH pH 7.7) and incubated in 1 mg/ml of collagenase. Once dissociated, oocytes were injected with 50 ng (10 nl) of wild type or His-Arpp19 mutant forms. After 1 h, PG was added at a final concentration of 37 µM in Merriam buffer (10 mM Hepes, 0.82 mM MgSO_4_, 88 mM NaCl, 0,33 mM Ca(NO_3_)_2_, 1 mM KCl, 0,41 mM CaCl_2_ pH 7.4) and GVBD (assessed by pigment rearrangement in the animal pole) was recorded. A sample of prophase I-arrested oocytes or germinal vesicle at the initial point of the experiment, as well as mature oocytes at the end of the experiment were collected, homogenized at 4 °C in extraction buffer (80 mM β-glycerophosphate, 20 mM EGTA, 15 mM MgCl_2_, 2 mM EDTA), centrifuged 15,000 × *g* for 10 min at 4 °C and lysates used for western blotting.

Kinase-inactivated egg extracts were obtained from laid eggs that were dejellied on 2% cysteine solution pH 7.8, transferred into MMR solution and washed twice with XB Buffer (50 mM sucrose, 0.1 mM CaCl_2_, 1 mM MgCl_2_, 100 mM KCl, HEPES pH 7.8). Eggs were subsequently centrifuged twice for 20 min at 10,000 × *g* and the cytoplasmic fractions recovered, supplemented with RNAse (10 μg/ml final concentration) and dialysed vs. a solution of 50 mM Tris pH 7.7, 100 mM NaCl overnight to eliminate ATP. Upon dialysis, extracts were ultracentrifuged for 50 min at 300,000 × *g* and supernatant recovered for use.

CSF extracts were obtained from dejellied eggs and washed with XB buffer with 5 mM EGTA, to prevent activation. Eggs were then centrifuged twice for 20 min at 10,000 × *g* and the cytoplasmic fraction recovered for use. mRNAs encoding the constitutive form of rat CamKII comprising amino acids 1–290 were transcribed ‘in vitro’ from pCS2-(1–290) rCamK2 plasmid with the SP6 RNA polymerase and translated in reticulocyte lysates. To promote meiotic exit, 2 μl of constitutive CamKII-translated reticulocyte lysates were then supplemented to 20 μl CSF extracts^14^.

### Immunoprecipitation/immunodepletion

Immunoprecipitations/immunodepletions were performed using 10 μl of extracts, 10 μl of protein G-magnetic Dynabeads (Life Technologies) and 2 μg of each antibody. Antibody-linked beads were washed twice with XB buffer, twice with Tris 50 mM pH 7.5 and incubated for 15 min at room temperature (RT) with 10 μl of *Xenopus* egg extracts. For immunodepletion, the supernatant was recovered and used for subsequent experiments.

Four rounds of immunoprecipitation were required to fully deplete endogenous Arpp19 from CSF extracts.

For B55 depletion, 1.5 ml of ATP-devoid extracts were adjusted at a final concentration of 400 mM NaCl and loaded on a 1.5 ml TALON Superflow Metal Affinity Resin column pre-bound with of 2 mg of His-Arpp19 wild-type purified protein. The flowthrough was then collected in different fractions, analysed by western blotting for B55 levels, aliquoted and frozen at −80 °C until use.

For immunoblotting, 0.75 μl of egg extract was subjected to SDS-polyacrylamide gel electrophoresis (PAGE), transferred to Protran nitrocellulose (Protran, Amersham) or Immobilon membranes (Millipore), and upon blocking using TBST/5% milk or TBST/5% bovine serum albumin (BSA) (when phospho-antibodies were used) incubated with the indicated antibodies.

When experiments to assess the capacity of Arpp19 mutants to promote mitotic entry were performed, 0.2 μg of exogenous Arpp19 and 50 ng of K72M Gwl were added in 20 μl of Arpp19-depleted egg extracts and a sample of 2 μl was recovered and used for western blotting.

### Plasmids

For (48–120) Arpp19 mutant, a 6His tag was subcloned in the pMal-C2X plasmid in the AscI-FseI cloning site. Arpp19 was subsequently subcloned in this double maltose-binding protein (MBP)-6His Tag vector in the BamHI–HindIII site.

*Xenopus* ENSA was amplified from *Xenopus* ovary cDNAs and subcloned into the pET15-6His vector in the NdeI–BamHI cloning site.

Human PKA and Prc1 clones were obtained from human ORFeome^25^ version 8 and subcloned in a pDON gateway vector. cDNAs were subsequently subcloned by Gateway in a pET15b vector.

pCMVsport6–*Xenopus* B56γ was obtained from RZPD Deutsches Ressourcenzentrum für Genomforschung GmbH, amplified by PCR and subcloned at the BamHI–SalI site of pGEX4T2.

Primers used for site-directed mutagenesis were purchased from Eurogentec and are detailed in the Supplementary Table 1.

### Antibody production

Polyclonal rabbit antibodies against Cter *Xenopus* Cdk1 peptide NH2-CLDKSSLPANQIR-COOH were used for Cdk1 immunoprecipitation. This peptide was covalently coupled to thyroglobulin using sulfo-MBS for immunization. Polyclonal Abs were then purified using the Cter Cdk1 peptide covalently coupled to BSA-sepharose beads using CNBR-sepharose.

Phospho-S109/S113 Arpp19 antibody was produced using phosphorylated S113 peptide (NH2-CLPQRKP**S(p)**LVASKL-COOH). This peptide was covalently coupled to thyroglobulin protein and injected into one rabbit. Rabbit polyclonal antibodies were then affinity purified against the phosphorylated S109/S113 Arpp19 peptide covalently coupled to BSA-sepharose beads.

For anti-*Xenopus* B56γ antibodies, GST–B56γ was purified and used to immunize rabbits. Immune serum was first pre-cleared of the anti-GST antibodies in a GST-immobilized column and were subsequently affinity purified on immobilized GST–B56γ columns.

### Antibodies

The antibodies used in this study are the following: Rabbit Polyclonal anti-Human Gwl^5^, Rabbit Polyclonal Phospho-Cdc2 (Tyr15) (Cell Signaling Technology Cat#9111), Rabbit Polyclonal anti-*Xenopus* Arpp19^14^, Rabbit Polyclonal anti-PP2A/B55δ (Cell Signaling Technology Cat#2290), Rabbit Polyclonal anti-*Xenopus* Cdc27^26^, Rabbit Polyclonal anti-*Xenopus* Cyclin B2^27^, Rabbit Polyclonal anti-*Xenopus* Cdk1^28^, Rabbit Monoclonal PhosphoThr320 of PP1 (Abcam Cat#62334), Mouse Monoclonal Phospho-Erk (Cell Signaling Cat# 9106 S), Rabbit Polyclonal anti-phosphorylated Arpp19 (S67) (Cell Signaling Cat#5240 S), Rabbit Polyclonal anti-PRC1 (Santa Cruz Cat# 376982), Goat Polyclonal anti-phosphorylated PRC1 (T481) (Santa Cruz Cat#11768), Mouse Monoclonal anti-PP2A (C) subunit-α (isoform Merck Millipore Cat#05-42), Rat Polyclonal PP2A (A) subunit (Cell Signaling Cat#2260), Mouse Monoclonal anti-PP1 kindly gifted by Dr M Bollen and used in Ma et al.^14^, Anti-PP4c (Bethyl Cat#A300-835A), Anti-PP6 (Santa Cruz Cat#393294), Goat anti-rat IgG-horseradish peroxidase (HRP) (Santa Cruz Cat#2006), HRP-conjugated anti-Rabbit secondary antibodies (Cell Signalling Technology Cat#7074), Donkey anti-goat IgG-HRP (Santa Cruz Cat# sc-2020), Rabbit Polyclonal anti-phosphorylated Arpp19 (S109/S113) (this study), and Rabbit Polyclonal anti-PP2A (B56) γ-subunit (this study).

Antibodies were diluted at 1/1000.

### ‘In vitro’ phosphorylation

Phosphorylation of Arpp19 on S67/S71 or of ENSA on S67 by Gwl was induced by using GST-K72M hyperactive mutant form of Gwl purified from SF9 cells. For ‘in vitro’ phosphorylation reaction, 6His-Arpp19 protein and GST-K72M Gwl kinase were mixed at a final concentration of 0.5 μg/μl and 45 ng/μl, respectively, in a reaction buffer (1 mM ATP, 2 mM MgCl_2_ and Tris 50 mM).

When thio-phosphorylation was performed, regular ATP was substituted by a final concentration of 1 mM of ATP^γs^. For radio-labelling of 6His-Arpp19 on S67/S71, 6His-Arpp19 and GST-Gwl K72M were mixed at a final concentration of 0.5 μg/μl and 45 ng/μl, respectively, in a reaction buffer containing 200 μM final ATP concentration and ATPγ^33^P at a specific activity of 5 μCi/nMol in 2 mM MgCl_2_, 100 mM NaCl and 50 mM Tris pH 7.5.

For phosphorylation of Arpp19 on S109/S113 or ENSA on S109 by PKA, a final concentration of 0.5 μg/μl of Arpp19/ENSA and of 50 ng/μl of 6His-PKA catalytic subunit purified from His-tag column in a reaction buffer containing 1 mM ATP, 2 mM MgCl_2_, 100 mM NaCl and 50 mM Tris pH 7.5 was used.

For phosphorylation of 6His-Prc1 on T481, Cdk1 immunoprecipitate was used. One microgram of 6His-human Cyclin A was mixed with 100 μl of CSF extracts during 30 min and subsequently supplemented with 50 μl of protein G-magnetic Dynabeads pre-linked with 10 μg of *Xenopus* Cdk1 C terminus antibodies. After 45 min incubation, the beads were washed three times with 500 mM NaCl, 50 mM Tris pH 7.5, twice with 100 mM NaCl, 50 mM Tris pH 7.5 and finally resuspended with 100 μl of reaction buffer containing 1 mM ATP, 2 mM MgCl_2_, 100 mM NaCl and 50 mM Tris pH 7.5. 6His-Prc1 was then added to the beads at a final concentration of 1.5 μg/μl.

All ‘in vitro’ phosphorylation reactions were incubated for 1 h at RT, aliquoted and frozen at −80 °C until use.

### Dephosphorylation reactions in ATP-devoid interphase egg extracts

When either S67/S71 or S109/S113 Arpp19, S67 or S109 ENSA, or T481 Prc1 dephosphorylation was checked by western blotting in ATP-devoid interphase *Xenopus* egg extracts, 1 μl of the corresponding ‘in vitro’ phosphorylation mix was diluted with 9 μl of Tris 50 mM–10 mM EDTA buffer and supplemented with 10 μl of ATP-devoid interphase extracts. Final concentration of the mix was adjusted to 300 mM NaCl with a solution of 5 M NaCl Tris 50 mM pH 7.5 and a sample of 2 μl was recovered at the indicated time points. From the 2 μl sample, 0.7 μl were used to check Arpp19/Prc1 amounts and the rest to assess phosphorylation of the indicated residues using specific phospho-antibodies. Time point 0 min was prepared by adding separately 1 μl of extract and 1 μl of phosphorylated substrate directly in Laemmli buffer.

When the impact of Arpp19/ENSA phosphorylation of S67/S71 on S109/S113 Arpp19 was checked in ATP-devoid interphase extracts, 2 μl of ‘in vitro’-phosphorylated Arpp19 by PKA sample and 2 μl of the ‘in vitro’-phosphorylated Arpp19 by Gwl sample were mixed with 6 μl of Tris 50 mM–10 mM EDTA buffer and 20 μl of ATP-devoid interphase extracts, and incubated at RT. Four microliters of sample were recovered at the indicated time points and were used for SDS-PAGE and western blotting.

For the analysis of the effect of S67/S71 phosphorylation of Arpp19 on T481 Prc1 dephosphorylation, 1 μl of ‘in vitro’ phosphorylated Prc1 by Cyclin A/Cdk was mixed with 1 μl of ‘in vitro’ phosphorylated Arpp19 by GwlK72M, 8 μl of Tris 50 mM–10 mM EDTA and 10 μl of ATP-devoid interphase extracts and incubated at RT. Two microliters of the mix were recovered at the indicated time points and used for SDS-PAGE and western blotting.

When dephosphorylation of Arpp19 mutants was assessed in ATP-devoid extracts by autoradiography, 1 μl of the ‘in vitro’ ^33^P-labelled GwlK72M-phosphorylated Arpp19 mutants were supplemented with 9 μl of Tris, 50 mM EDTA, 10 mM buffer and mixed with 10 μl of ATP-devoid extracts an incubated at RT.Two-microlitre samples were then recovered at the indicated time points and submitted to western blot and autoradiography. In the case of B55-depleted ATP-devoid interphase extracts and due to the fact that a residual B55 was occasionally observed in the extracts, 0.5 μg of non-radioactive wild-type S67/S71 pre-phosphorylated Arpp19 was supplemented to the reaction volume, to ensure a full inactivation of PP2A-B55. Samples were then recovered at the indicated times and submitted to western blot and autoradiography.

Dephosphorylation reactions with purified PP2A-B55 phosphatase were performed as for non-depleted ATP-devoid extracts, except that purified preparation of PP2A-B55 was diluted four times with 400 mM Nacl Tris 50 mM pH 7.5.

### Gel filtration

Three millilitres of ATP-devoid interphase extracts were loaded into a Hiload 16/60 Superdex 200 (GE Healthcare, Life Science) equilibrated with a Tris 50 mM pH 7.5, 100 mM NaCl buffer and eluted at a flow rate of 0.9 ml/min into fractions of 1.8 ml. Arpp19 S109/S113 and S67/S71 dephosphorylation activities were determined by mixing 5 μl of each elution fraction and 2 μl of ‘in vitro’ phosphorylated Arpp19 by either Gwl or PKA diluted 20 times in Tris 50 mM pH 7.5, 100 mM NaCl, 10 mM EDTA. 10 minutes incubation for phospho-Arpp19 S109/S113 and 20 min for phospho-Arpp19 S67/S71 were used.

### H1 kinase activity

CSF extract samples (1 μl) were frozen at the indicated times during the experiment. When H1 kinase activity was performed, samples were thawed by the addition of 19 μl of phosphorylation buffer including 2 μCi [γ^33^P] ATP and incubated for 10 min at RT. Reactions were stopped by Laemmli sample buffer addition and used for SDS-PAGE and autoradiography.

### Mutagenesis

Deletions and single or double point mutations of Arpp19 were performed using Pfu ultra II fusion DNA polymerase. Oligonucleotides were purchased from Eurogentec and are detailed in the Supplementary Material, Table 1. A pMA-T vector encoding for a DNA in which the DSG domain of Arpp19 sequence was exchanged by the cassette domain (D2-D1 mutant) was synthesized by Geneart (Thermofisher) and subcloned into Pet-15 6His vector.

### Protein purification

6His-*Xenopus* Arpp19 (isoform 1, accession number XP_018106497.1^14^, 6His-Xenopus ENSA (accession number NM_001086605.1), 6His-human Prc1 and 6His-Rat Catalytic Subunit of PKA were produced in *Escherichia coli* and purified using TALON Superflow Metal Affinity Resin. Due to the insolubility of the histidine fusion protein for (48–120) N-terminal deletion mutant of Arpp19, a double-tagged MBP-His fusion protein was produced and purified using the histidine tag as reported above.

### PP2A-B55 purification

ATP-devoid interphase extracts (15 ml) were supplemented with NaCl to a final concentration of 400 mM NaCl and loaded into a 1 ml TALON Superflow Metal Affinity Resin column pre-bound with 10 mg of His-Arpp19 wild-type purified protein. After thoroughly washing with a Tris 50 mM pH 7.5, 400 mM NaCl buffer, elution was performed with 5 ml Imidazole 150 mM in Tris 50 mM pH 7.5, 100 mM NaCl. The eluted fraction was then loaded into a HiLoad 16/60 Superdex 200 column, equilibrated with a Tris 50 mM pH 7.5, 100 mM NaCl buffer and eluted at a flow rate of 0.9 ml/min into fractions of 1.8 ml. Fractions from 32 to 42 were pooled, diluted 1/3 in Tris 50 mM pH 7.5 and re-loaded into a MonoQ 5/50GL column GE Healthcare, Life Science) (Supplementary Fig. 5). After washing with a Tris 50 mM pH 7.5 buffer, elution was performed with a 0–800 mM NaCl linear gradient into fractions of 0.4 ml. After western blot analysis, fractions 15, 16 and 17 were pooled and used for dephosphorylation assays.

### PP2A-B55-binding assays

Twenty microliters of HisPur^TM^NiNTA Magnetic beads (Life Technologies) were washed with XB buffer and supplemented with 1 μg of 6His wild type or the mutant forms of Arpp19 and incubated at 21 °C for 20 min in continuous agitation. Beads were recovered, washed three times with XB buffer and supplemented with 25 μl of CSF extract. Upon 20 min of continuous mixing at 21 °C, beads were washed again for three times with RIPA buffer (10 mM NaH_2_PO_4_, 100 mM NaCl, 5 mM EDTA, 1% Triton X-100, 0.5% deoxycholate, 80 μM β-glycerophosphate, 50 mM NaF, 1 mM dithiothreitol), twice with HEPES 50 mM pH 7.4 and supplemented with Laemmli sample buffer, and used for western blotting with anti-Arpp19 and anti-B55 antibodies.

### Statistical analysis and reproducibility

Statistical analysis was performed by using GraphPad Software. Dephosphorylation experiments were repeated three times using different extracts. For each binding assay, sample size (*n*) is provided in figure legends. All experiments presented in this manuscript have been repeated at least three times.

## Supplementary information

Supplementary Information

## Data Availability

All data that support the findings of this study are available from the corresponding authors upon a reasonable request. There are no restrictions on data availability. Accession Codes are availables for *Xenopus* Arpp19 isoform 1 and *Xenopus* ENSA at: Xenopus Arpp19 Isoform 1: https://www-ncbi-nlm-nih-gov.insb.bib.cnrs.fr/protein/XP_018106497.1?report=genbank&log$=protalign&blast_rank=1&RID=7XZP051Y016 and Xenopus ENSA: https://www-ncbi-nlm-nih-gov.insb.bib.cnrs.fr/protein/NP_001080074.1?report=genbank&log$=protalign&blast_rank=1&RID=7UZM0P51016. For the PP2A structure, we used PDB 2IE4 from Xing et al.^24^. Source data are provided with this paper.

## Source data

Source data
