# Supplementary material for: The study of the determinants controlling Arpp19 phosphatase-inhibitory activity reveals an Arpp19/PP2A-B55 feedback loop
Source: Nat Commun. 2021 Jun 11;12:3565. doi: 10.1038/s41467-021-23657-0 (PMC8196004; doi:10.1038/s41467-021-23657-0)
Supplement: Supplementary file 1 — Supplementary Information [file 41467_2021_23657_MOESM1_ESM.pdf]

A

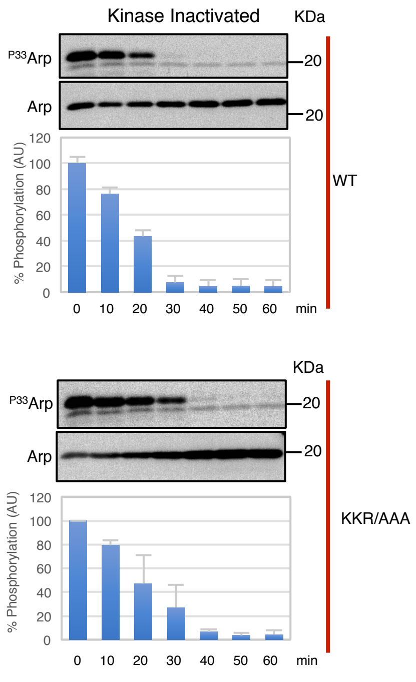

B

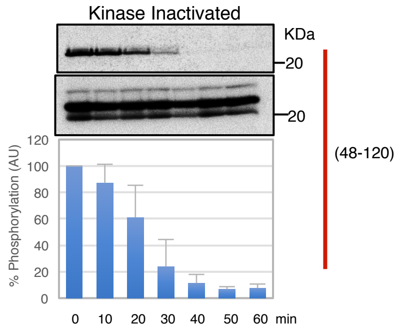

Supplementary Figure 1. Basic residues flanking the DSG motif positively modulate S71 dephosphorylation.

(A) Dephosphorylation of the wildtype and the KKR/AAA mutant form of Arpp19 was performed three times in kinase-inactivated extracts as indicated in material and methods at different kinetics between 0 and 60 minutes. Levels of Arpp19 and S71 phosphorylation were analysed by western blot and autoradiography and quantified by ImageJ. The mean percentage of the ^{33}P-Arpp19/ Arpp19 ratio remaining at each time-point respect to time 0 min was calculated and represented as bar graphs +/- SD; n=3 biological independent samples.

(B) As for (A) except that the (48-120) mutant form of Arpp19 was used. The mean percentage of the ^{33}P-Arpp19/ Arpp19 ratio remaining at each time-point respect to time 0 min was calculated and represented as bar graphs +/- SD; n=3 biological independent samples.

A

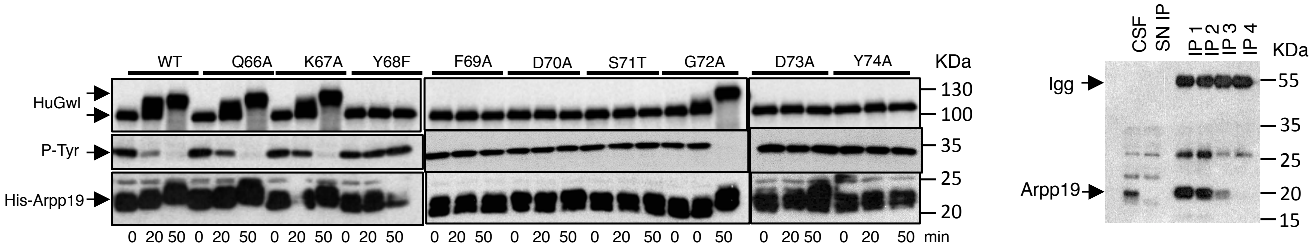

B

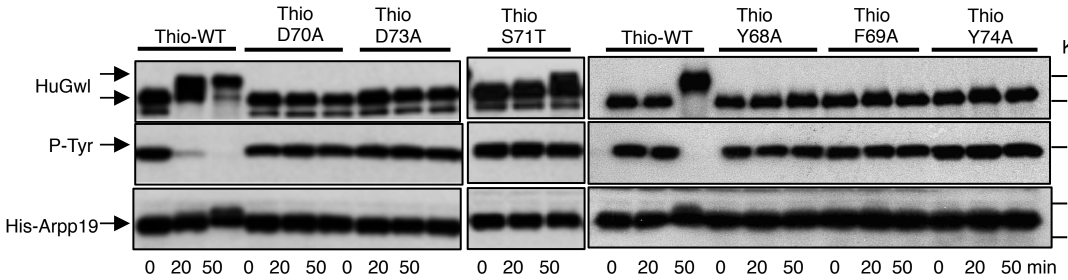

Supplementary Figure 2. Aromatic and acidic residues flanking S71 Gwl site of Arpp19 are essential to promote mitotic entry to Arpp19 devoid extracts.

(A) The wildtype or the indicated mutants of Arpp19 were supplemented simultaneously with a trace amount of human GwlK72M and the capacity of this protein to promote mitotic entry in Arpp19-depleted extracts analyzed. The phosphorylation of human ectopic GwlK72M together with the phosphorylation of Tyr 15 of Cdk1 and the levels of ectopic Arpp19 wildtype or mutant proteins were measured by western blot (left panels). In order to fully deplete Arpp19, four rounds of immunoprecipitation were performed in CSF extracts. The amount of Arpp19 remaining upon four immunoprecipitations is shown (right panel). Igg: G Immunoglobulines. SN IP: Supernatant of the fourth immunoprecipitation.

(B) As for (B) except that the wildtype or the mutant forms Arpp19 were thio-phosphorylated *"in vitro"* by GwlK72M before being added to the Arpp19-depleted extracts.

Experiments supporting the data of this figure were performed at least three times.

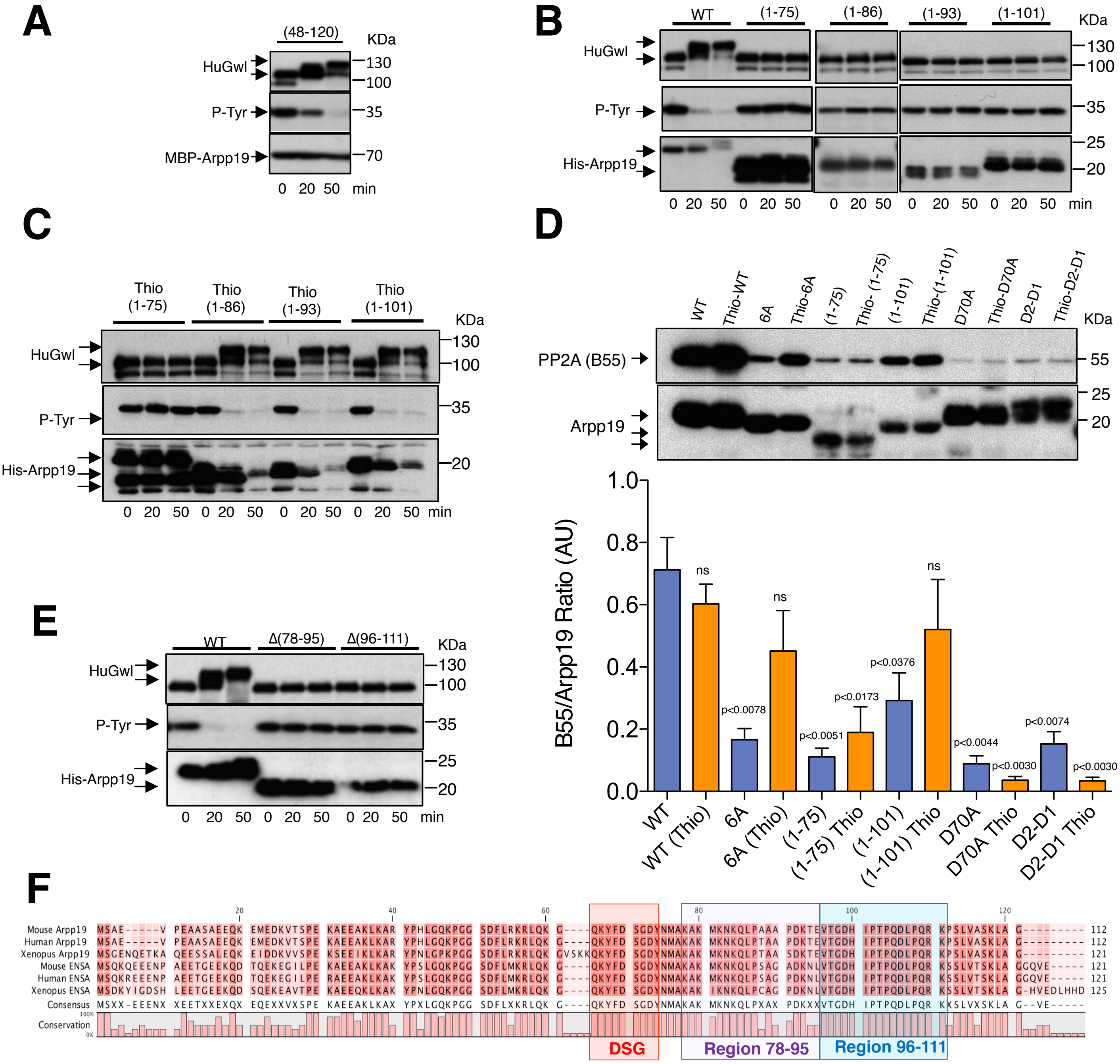

Supplementary Figure 3. Arpp19 PP2A-B55 inhibitory activity depends on a specific sequence of the "cassette" motif as well as on a minimal distance between this sequence and the DSG motif.

(A) The capacity of the (48-120) Arpp19 mutant to restore mitosis in Arpp19-depleted extracts is determined.

(B), (C) and **(E)** as for (A) except that the indicated mutants were thio-phosphorylated or not as indicated and tested.

(D) The wildtype and the indicated Arpp19 mutants were thio-phosphorylated or not and submitted to His-pulldown as indicated in material and methods except for the use of a less stringent washing of beads with XB buffer. Assays were repeated three times, B55 bound to the beads was measured and corrected by the levels of Arpp19. Represented is the mean B55/Arpp19 ratio +/- SD. Two-tailed unpaired Student t tests were performed in each pulldown to determine statistical relevance. p versus wildtype Arpp19. ns: non-significant.

(F) Depicted are sequence homology of Arpp19 between different species. Regions 78-95 and 96-11 as well as the DSG motif are indicated with violet, blue and red squares respectively. Note that region 96-111 is highly conserved. All the experiments were performed by triplicate.

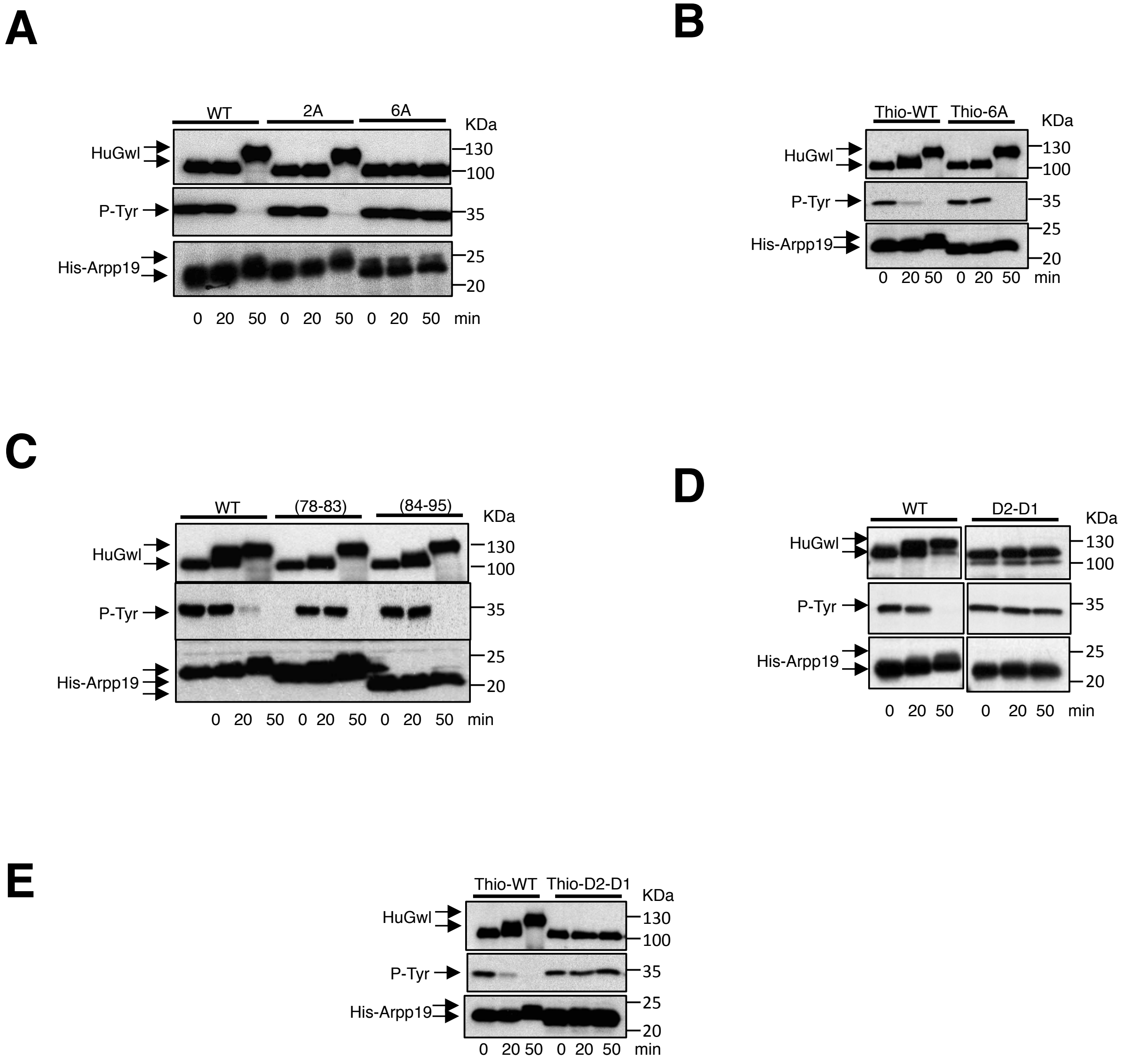

Supplementary Figure 4. Arpp19 PP2A-B55 inhibitory activity depends on a specific sequence of the "cassette" motif as well as on a minimal distance between this sequence and the DSG motif.

(A) The capacity of the 2A and 6A Arpp19 mutants to restore mitosis in Arpp19-depleted extracts is determined.

(B) 6A Arpp19 mutant form was thio-phosphorylated and its capacity to restore mitosis in Arpp19-depleted extracts tested.

(C) and **(D)** as for (A) except that the indicated mutants were tested.

(E) Rescue assays were performed as for the other mutants except that a thio-D2-D1 phosphorylated form was used.

Experiments supporting the data of this figure were performed at least three times.

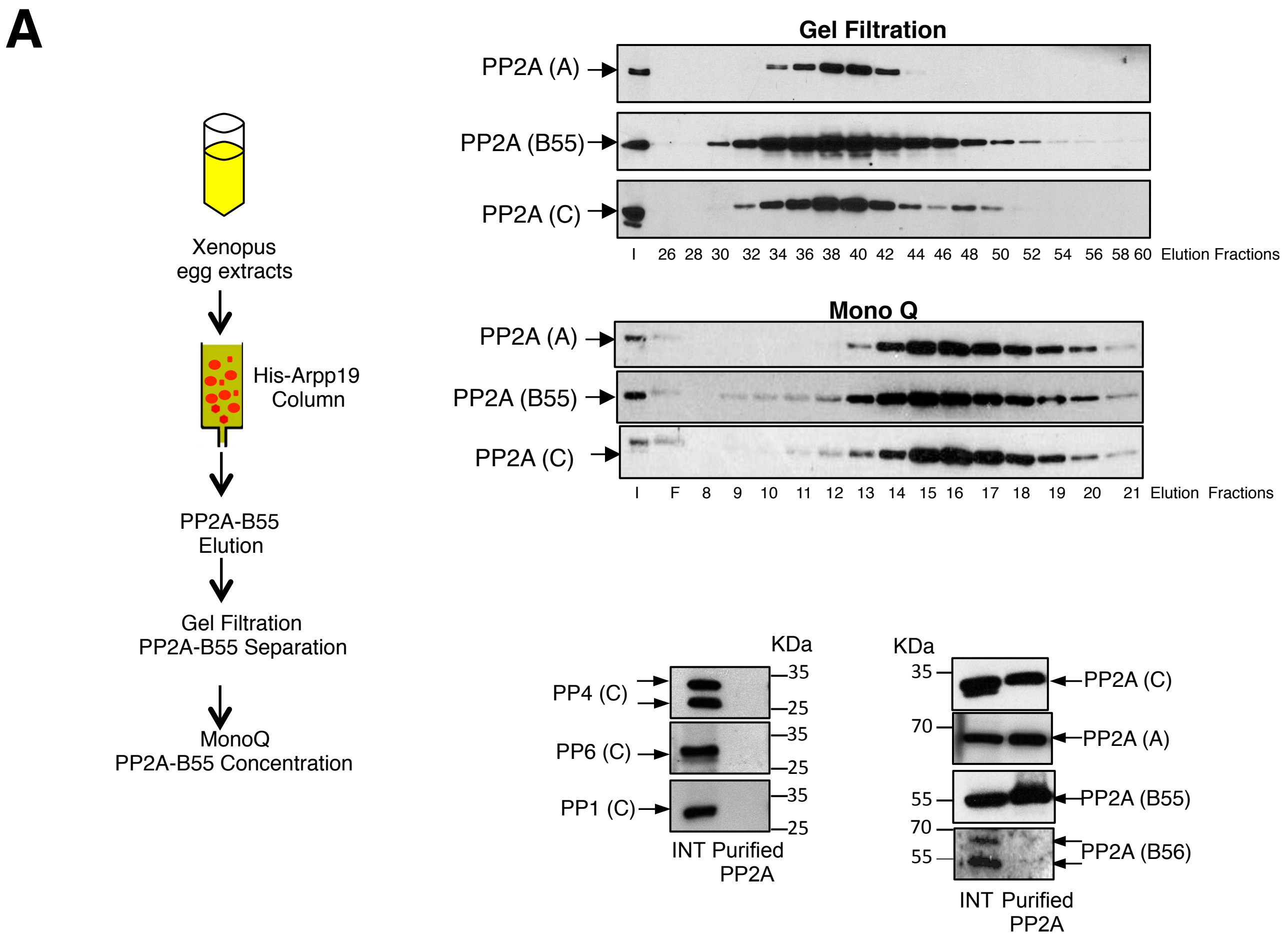

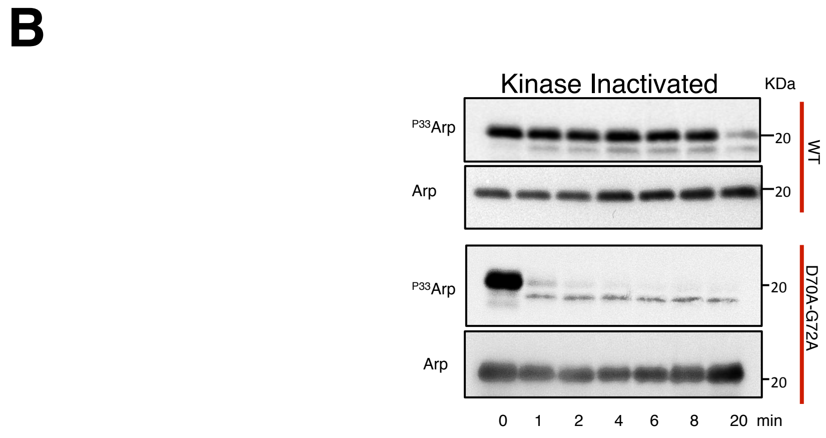

Supplementary Figure 5. Schematic representation of the procedure used to purify PP2A-B55 phosphatase form Xenopus egg extracts.

(A) Shown are the amount of PP2A A, B55 and C subunits present in the elution fractions of gel filtration and MonoQ columns. The levels of PP4, PP6, PP1, and A, C, B55 and B56 subunits of PP2A were also examined in the final mix of fractions 15, 16 and 17 representing the pick of purified PP2A-B55 used for the rest of the study. I: Interphase extracts; F: flow through.

(B) The dephosphorylation of S67/S71 of the wildtype or the D70A-G72A double mutant of Arpp19 were assayed in kinase-inactivated extracts and revealed by autoradiography. The amount of this Arpp19 mutant form in each sample is also shown. All the experiments were repeated at least three times.

REAGENT OR RESOURCE	SOURCE	IDENTIFIER
**BACTERIAL AND VIRUS STRAINS**		
BL21DE3 Competent E.Coli	New England Biolabs	Cat#C2527H
DH5α E. Coli	New England Biolabs	Cat#C2987I
**CHEMICALS, PEPTIDES AND RECOMBINANT PROTEINS**		
[gamma-P33] ATP	HARTMANN ANALYTIC	Cat#SRF-301
Pfu ultra II fusion DNA polymerase	Agilent	Cat#600670
ATPγS	Sigma	Cat#A1388
TALON Superflow Metal Affinity Resin	Takara	Cat#635506
PVDF transfer membrane	Millipore	Cat#88518
Protan Nitrocellulose membran	Amersham	Cat#GE10600016
Dynabeads protein G	Life Technologies	Cat#10004D
Histone H1	Sigma	Cat#14–155
BSA	Sigma	Cat#A7906
His-PureTMNiNTA magnetic beads	Life Technologies	Cat#88832
CNBr-activated sepharose 4B	GE Healthcare	Cat#17–0430–01
Amylose Resin High Flow	Biolabs	Cat#E80225
Sulfo-MBS	Thermo Scientific	Cat#22312
HiLoadTM16/600 Superdex	Thermo Scientific	Cat#28–9893–35
MonoQ 5/50 GL	GE-Healthcare	Cat#GE17–5166–01
Recombinant GST-Human Greatwall K72M mutant	Vigneron et al. 2011	N/A
GST-Xenopus B56 gamma	This study	N/A

REAGENT OR RESOURCE	SOURCE	IDENTIFIER
**OLIGONUCLEOTIDES**		
Forward and reverse primers for Arpp19 K36A/K38A/R40A:<br>5' GAAGTCAGAGGAGATAGCGTTAGCAGCAGCGTATCCTAACCTCGG 3'<br>5' CCGAGGTTAGGATACGCTGCTGCTAACGCTATCTCCTCTGACTTC 3'	Eurogentec	This Study
Forward and reverse primers for Arpp19 Q66A:<br>5'GGCGTAAGTAAAAAGGCAAAATATTTTGACTCTGGG 3'<br>5' CCCAGAGTCAAAATATTTTGCCTTTTTACTTACGCC 3'	Eurogentec	This Study
Forward and reverse primers for Arpp19 K67A:<br>5' GGCGTAAGTAAAAAGCAAGCATATTTTGACTCTGGGGAC 3'<br>5' GTCCCCAGAGTCAAAATATGCTTGCTTTTTACTTACGCC 3'	Eurogentec	This Study
Forward and reverse primers for Arpp19 Y68A:<br>5' GTAAGTAAAAAGCAAAAAGCTTTTGACTCTGGGGAC 3'<br>5' GTCCCCAGAGTCAAAAGCTTTTTGCTTTTTACTTAC 3'	Eurogentec	This Study
Forward and reverse primers for Arpp19 F69A:<br>5' GTAAGTAAAAAGCAAAAATATGCTGACTCTGGGGACTACAT 3'<br>5' ATTGTAGTCCCCAGAGTCAGCATATTTTTGCTTTTTACTTC 3'	Eurogentec	This Study
Forward and reverse primers for Arpp19 D70A:<br>5' AAAGCAAAAATATTTTgccTCTGGGGACTACAATATG 3'<br>5' CATATTGTAGTCCCCAGAggcAAAATATTTTTGCTTT 3'	Eurogentec	This Study

REAGENT OR RESOURCE	SOURCE	IDENTIFIER
**OLIGONUCLEOTIDES**		
Forward and reverse primers for Arpp19 S71T:<br><br>5' GGCCAAAAATATTTTGACACTGGGGA CTACAATATGGC 3'<br>5' GCCATATTGTAGTCCCCAGTGTCAAAA TATTTTTGGCC 3'	Eurogentec	This Study
Forward and reverse primers for Arpp19 G72A:<br><br>5' CAAAAATATTTTGACTCTGCGGACTAC AATATGGCTAAA 3'<br>5' TTTAGCCATATTGTAGTCCGCAGAGTC AAAATATTTTTG 3'	Eurogentec	This Study
Forward and reverse primers for Arpp19 D73A:<br><br>5' TATTTTGACTCTGGGGCCTACAATATG GCTAAA 3'<br>5' TTTAGCCATATTGTAGGCCCCAGAGTC AAAATAG 3'	Eurogentec	This Study
Forward and reverse primers for Arpp19 Y74A:<br><br>5' TATTTTGACTCTGGGGACGCCAATATG GCTAAAGCA 3'<br>5' TTTAGCCATATTGTAGGCCCCAGAGTC AAAATAG 3'	Eurogentec	This Study
Forward and reverse primers for Arpp19 (48-120):<br><br>5' CGCGGATCCAGAAAGCGACTTCAGAA AGGCG 3'<br>5' CCCAAGCTTTCAGCCAGCCAGTTTGCT TGCG 3'	Eurogentec	This Study
Forward and reverse primers for Arpp19 (1-75):<br><br>5' GAC-TCT-GGG-GAC-TAC-AAT-TAG-GCT-AAA-GCA-AAG-ATG 3'<br>5' CAT-CTT-TGC-TTT-AGC-CTA-ATT-GTA-GTC-CCC-AGA-GTC 3'	Eurogentec	This Study

REAGENT OR RESOURCE	SOURCE	IDENTIFIER
**OLIGONUCLEOTIDES**		
Forward and reverse primers for Arpp19 (1-86):<br>5' GATGAAGAACAAGCAACTGTAAACAG CTGCATCTGATAAA3'<br>5' TTTATCAGATGCAGCTGTTTACAGTTG CTTGTTCTTCATC 3'	Eurogentec	This Study
Forward and reverse primers for Arpp19 (1-93):<br>5' CAACAGCTGCATCTGATAAATAGGAG GTTACGGGTGATCAT 3'<br>5' ATGATCACCCGTAACCTCCTATTTATCA GATGCAGCTGTTG 3'	Eurogentec	This Study
Forward and reverse primers for Arpp19 (1-101):<br>5' GTTACGGGTGATCATATTTAGACGCCA CAAGACCTCCCT 3'<br>5' GAGGGAGGTCTTGTGGCGTCTAAATA TGATCACCCGTAAC 3'	Eurogentec	This Study
Forward and reverse primers for Arpp19 D(78-95):<br>5' CTCTGGGGACTACAATATGGCTGTTAC GGGTGATCATATTCC 3'<br>5' GGAATATGATCACCCGTAACAGCCAT ATTGTAGTCCCCAGAG 3'	Eurogentec	This Study
Forward and reverse primers for Arpp19 D(96-111):<br>5' GCTGCATCTGATAAAACGGAGCCGTC TCTCGTTGCAAGCA 3'<br>5' TGCTTGCAACGAGAGACGGCTCCGTTT TATCAGATGCAGC 3'	Eurogentec	This Study
Forward and reverse primers for Arpp19 2A:<br>5' CGGGTGATCATATTCCTGCGGCACAA GACCTCCCTCAAAG 3'<br>5' CTTTGAGGGAGGTCTTGTGCCGCAGG AATATGATCACCCG 3'	Eurogentec	This Study

REAGENT OR RESOURCE	SOURCE	IDENTIFIER
**OLIGONUCLEOTIDES**		
Forward and reverse primers for Arpp19 6A:<br>5’ CATATTCCTGCGGCACAAGCCGCCGCTGCAAGGAAACCGTCTCTC 3’<br>5’ GAGAGACGGTTTCCTTGCAGCGGCGGCTTGTGCCGCAGGAATATG 3’	Eurogentec	This Study
Forward and reverse primers for Arpp19 D(78-83):<br>5’ TCTGGGGACTACAATATGGCTCAACTGCCAACAGCTGCATCT 3’<br>5’ AGATGCAGCTGTTGGCAGTTGAGCCATATTGTAGTCCCCAGA 3’	Eurogentec	This Study
Forward and reverse primers for Arpp19 D(84-95):<br>5’ GGCTAAAGCAAAGATGAAGAACGTTACGGGTGATCATATTCC 3’<br>5’ GGAATATGATCACCCGTAACGTTCTTCATCTTTGCTTTAGCC 3’	Eurogentec	This Study
Forward and reverse primers for Arpp19 (D2-D1):<br>5’ GGTCGACAATGTCTGGAGAAAATCAGGAG 3’<br>5’ CGCGGATCCTCAGCCTTTAGCCATA 3’	Eurogentec	This Study
Forward and reverse primers for Arpp19 S113D:<br>5’CTCCCTCAAAGGAAACCGGATCTCGTTGCAAGCAAACTGG 3’<br>5’ CCAGTTTGCTTGCAACGAGATCCGGTTTCCTTTGAGGGAG 3’	Eurogentec	This Study

REAGENT OR RESOURCE	SOURCE	IDENTIFIER
**RECOMBINANT DNA**		
Lorca et al. 1993	Lorca et al. 1993	N/A
pET7F1-human Cyclin A	Generous gift of G Draetta.; Lorca et al.1992	N/A
pET15-6His-Xenopus Arpp19	Ma et al. 2016	N/A
pFastBac-GST-hGwlK72M	Vigneron et al. 2011	N/A
pET15-6His-Xenopus ENSA	This Study	N/A
pMalCX2-His-Arpp19	This Study	N/A
pET15b-human PKA	This study	N/A
pCMVsport6–Xenopus B56 gamma 3’	This study	N/A
pET15b-human PRC1	This Study	N/A

REFERENCES:

Lorca, T. *et al.* Cyclin A-cdc2 kinase does not trigger but delays cyclin degradation in interphase extracts of amphibian eggs. *Journal of cell science* **102 (Pt 1)**, 55–62 (1992).

Lorca, T. *et al.* Calmodulin-dependent protein kinase II mediates inactivation of MPF and CSF upon fertilization of Xenopus eggs. *Nature* **366**, 270–3 (1993).

Vigneron, S. *et al.* Characterization of the Mechanisms Controlling Greatwall Activity. *Molecular and Cellular Biology* **31**, 2262–2275 (2011).

Ma, S. *et al.* Greatwall dephosphorylation and inactivation upon mitotic exit is triggered by PP1. *Journal of cell science* **129**, 1329–39 (2016).
